# Nontargeted Metabolomics of *Streptomyces* Sourced from Thailand Reveals the Presence of Bioactive Metabolites

**DOI:** 10.1021/acsomega.5c00669

**Published:** 2025-03-11

**Authors:** Yuwathida Sunghanghwa, Atchara Paemanee, Kiep Minh Do, Mathurin Meethangdee, Michaela Plechatá, Wasu Pathom-Aree, Chuchard Punsawad, Hiroyuki Morita, Sithichoke Tangphatsornruang, Zdenek Kamenik, Amit Jaisi

**Affiliations:** † College of Graduate Studies, Walailak University, Thasala, Nakhon Si Thammarat 80160, Thailand; ‡ National Center for Genetic Engineering and Biotechnology, National Science and Technology Development Agency, Khlong Nueng, Pathum Thani 12120, Thailand; § Faculty of Pharmacy, Nam Can Tho University, Can Tho 900000, Vietnam; ∥ Department of Biology, Faculty of Science, Chiang Mai University, Chiang Mai 50200, Thailand; ⊥ University of Chemistry and Technology Prague, Technická 5, Prague 6-Dejvice, Prague 160 00, Czech Republic; # Institute of Microbiology of the Czech Academy of Sciences, Praha 14200, Czech Republic; ∇ School of Medicine, Walailak University, Thasala, Nakhon Si Thammarat 80160, Thailand; ○ Institute of Natural Medicine, University of Toyama, Toyama 930-0194, Japan; ◆ School of Pharmacy, Walailak University, Thasala, Nakhon Si Thammarat 80160, Thailand; ¶ Biomass and Oil Palm Center of Excellence, Walailak University, Thasala, Nakhon Si Thammarat 80160, Thailand

## Abstract

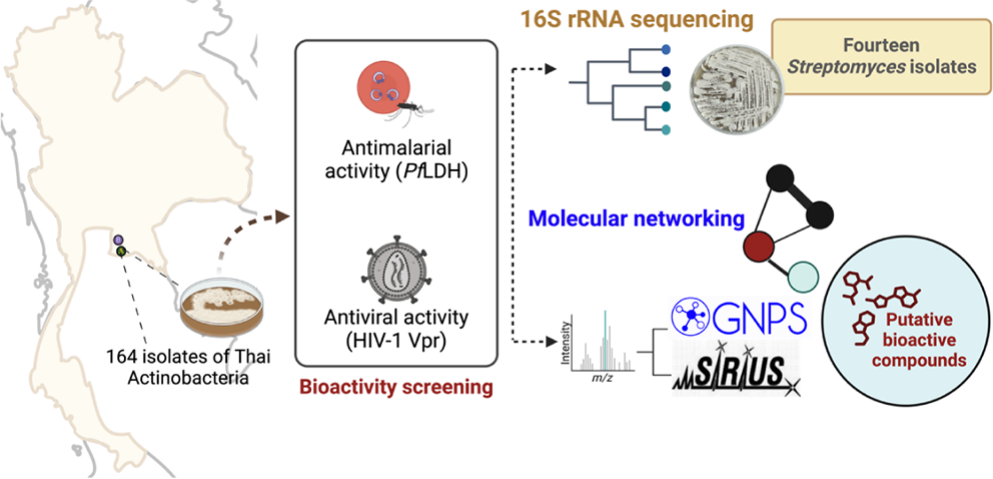

Actinobacteria are widely recognized as prolific producers of bioactive metabolites with diverse biological properties, yet they remain largely unexplored. In this study, we investigated the antimicrobial potential and chemical diversity of crude extracts from actinobacterial strains isolated from mangrove sediments collected in Chonburi and Chachoengsao provinces of Thailand. Taxonomic identification confirmed that these isolates belong to the genus *Streptomyces*. Notably, ten isolates, identified as *Streptomyces iranensis*, *Streptomyces yogyakartensis*, *Streptomyces cacaoi*, *Streptomyces ardesiacus*, *Streptomyces phaeoluteichromatogenes*, and *Streptomyces albiaxialis*, exhibited potent inhibitory activity against chloroquine-resistant *Plasmodium falciparum* K1 at concentrations <10 μg/mL. Among these, only *S. albiaxialis* displayed anti-human immunodeficiency virus-1 viral protein R (HIV-1 Vpr) activity in HeLa cells harboring the TREx plasmid encoding full-length Vpr (TREx-HeLa-Vpr cells). MS/MS-guided molecular networking analysis highlighted the metabolic complexity of the isolates, revealing a diverse array of distinct compounds. These included chymostatin B, geldanamycin, dehydroxynocardamine, ikarugamycin epoxide, kanchanamycin C, glochidone, bisucaberin, coproporphyrin III, futalosine, and various siderophores such as ferrioxamine B, desferrioxamine D2, desferrioxamine G, desferrioxamine E, desferrioxamine, desferrioxamine H, and ferrioxamine E. Moreover, guided by the potent antimalarial activity of strain S2-SC19, the compound elaiophylin was detected, isolated, and identified using analytical techniques. Remarkably, the compound exhibited potent antimalarial activity with an IC_50_ value of 0.002 ± 0.002 μg/mL against *P. falciparum* K1. Furthermore, genomic analysis revealed that strain S2-SC19 is most closely related to *Streptomyces asiaticus* DSM no. 41761. This study highlights Thai mangrove soil as a valuable source of bioactive compounds, including elaiophylin, and underscores the bioactive potential and chemical diversity of mangrove ecosystems as a rich, untapped reservoir of natural products.

## 1 Introduction

Actinobacteria, ubiquitous in diverse environments such as soil, marine habitats, and symbiotic associations, have long been praised for their prolific production of a broad spectrum of bioactive secondary metabolites.^1−3^ These compounds exhibit various biological activities, including antimalarial and antiviral properties.^4,5^ Historically, actinobacteria-derived natural products have played a pivotal role in the pharmaceutical industry, serving as the foundation for numerous clinically significant antibiotics, including streptomycin, erythromycin, and vancomycin.^1^

However, faced with the increasing emergence of multidrug-resistant pathogens, there is an urgent imperative to explore new avenues for antimicrobial discovery. One of the most intriguing aspects of actinobacteria is their capacity to produce multiple bioactive compounds from a single strain depending on the culture conditions, a phenomenon often termed “one strain, many compounds”.^6^ This inherent biosynthetic versatility offers numerous opportunities for drug discovery and development. Notably, among the diverse biological activities exhibited by actinobacteria-derived compounds, their potential as antimalarial and antiviral agents has garnered significant attention.

Malaria, caused by *Plasmodium* parasites transmitted through the bite of infected mosquitoes, remains a leading cause of morbidity and mortality worldwide. Despite substantial progress in malaria control efforts, the emergence and spread of drug-resistant strains, particularly those resistant to artemisinin-based combination therapies (ACTs), pose a formidable challenge.^7^ Actinobacteria-derived natural products hold promise as alternative or adjunctive therapies against drug-resistant malaria parasites, offering novel mechanisms of action and potential synergistic effects. The lactate dehydrogenase (LDH) enzyme found in *Plasmodium* species differs structurally and functionally from human LDH isozymes, making it a promising target for antimalarial drugs.^8^ LDH catalyzes the conversion of pyruvate to lactate in the final step of glycolysis, which is crucial for cellular energy production. In *P. falciparum*, survival hinges on hemozoin formation from hemoglobin degradation. Targeting *Pf*LDH’s active site with NADH competitors can inhibit hemozoin polymerization, leading to parasite death, akin to quinoline derivatives that complex with hematin.

Similarly, the persistent threat of human immunodeficiency virus (HIV) underscores the need for innovative approaches to combat drug resistance and enhance treatment outcomes.^9^ While antiretroviral therapy (ART) has revolutionized the management of HIV infection, the development of resistance mutations poses a significant barrier to long-term treatment success. The viral R protein (Vpr) has been widely studied in recent years as one of the potent targets for anti-HIV drug discovery. It is a viral accessory protein whose level is highly conserved in HIV type 1. It plays a regulatory role in various viral life cycle processes, e.g., regulation of nuclear import of the viral preintegration complex, viral transcription, cell cycle arrest, cell apoptosis, etc.^10^ Therefore, Vpr may be an attractive target in HIV drug development. Actinobacteria-derived compounds may offer novel targets or adjuvant therapies to overcome HIV drug resistance, prolonging existing treatment regimens’ efficacy and improving patient outcomes.

Molecular networking has been recognized as a powerful strategy for studying the chemical diversity of actinobacteria-derived natural products, enabling efficient dereplication of previously identified compounds.^11^ By utilizing advanced analytical techniques and computational tools, molecular relationships can be elucidated, allowing for the prioritization of lead compounds and accelerating the drug discovery process.

**Figure 1:**
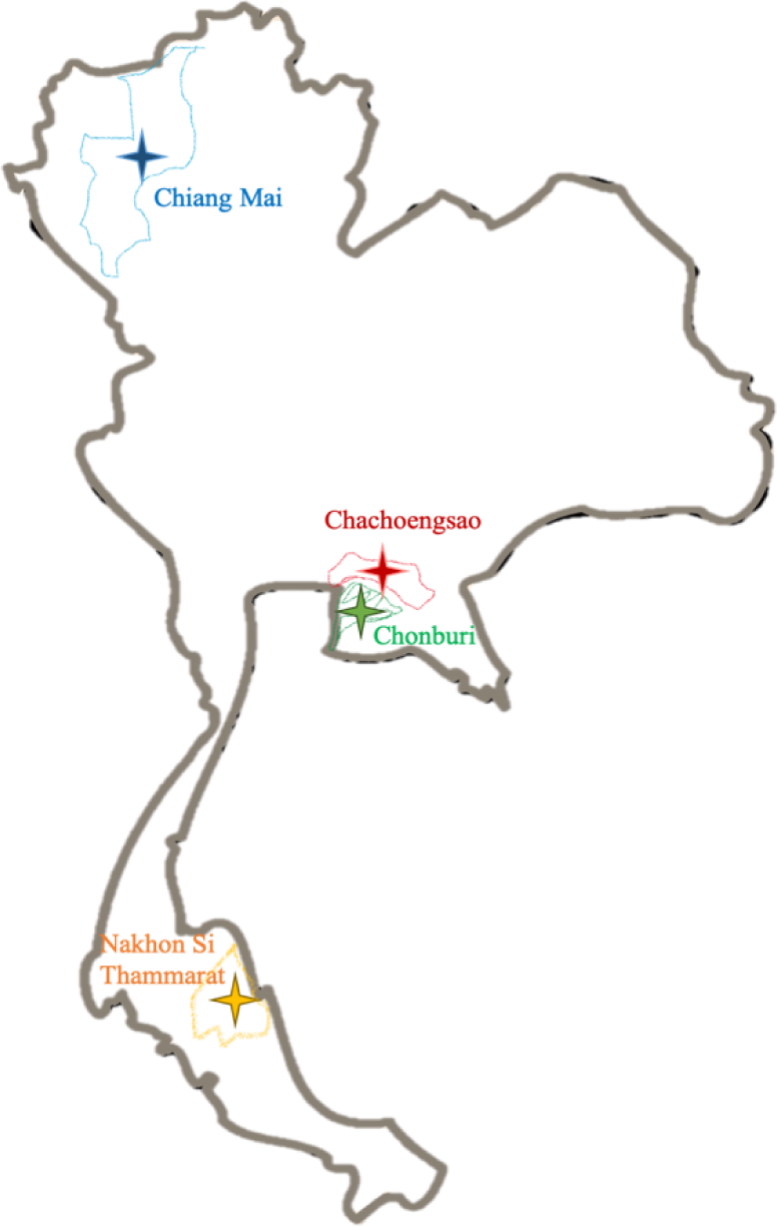
Collection sites of environmental samples for actinobacteria isolation in Thailand.

This study aimed to investigate the antiplasmodial and anti-HIV potential of natural products from mangrove derived actinobacteria (Figure 1), with a particular focus on their therapeutic potential against malaria. Through an in-depth examination of recent advancements and promising avenues of research, it aimed to drive efforts toward exploiting the Thai-rich reservoir of actinobacteria-derived natural products in the fight against infectious diseases and multidrug-resistant pathogens.

## 2 Results and Discussion

### 2.1 Antimalarial Activities of Mangrove Derived Actinobacterial Extracts

In total, 164 actinobacteria were isolated from two mangrove sediments collected from the Chonburi and Chachoengsao provinces in Thailand (Figure 1). The antimalarial activity of the 164 actinobacterial crude extracts was recorded based on the growth inhibition of the chloroquine-resistant *Plasmodium falciparum* K1 lactate dehydrogenase *(Pf*LDH). According to primary screening, at a concentration of 0.5 mg/mL crude extract, 15 actinobacterial isolates showed antimalarial activity with *Pf*LDH enzyme inhibition percentage above 80%, significantly (Table S1). These 15 actinobacterial isolates were further evaluated using a series of concentrations ranging from 0.31 ng/mL to 10 μg/mL to determine their IC_50_ values. Among the 15 positive isolates, 14 strains exhibited potent antimalarial activity, and one strain, however, showed a moderate inhibition of parasite growth, which was discarded for further investigation. Notably, three actinobacterial strains showed the highest inhibitory activity with IC_50_ values below 1 ng/mL, including strains 1-5-14, S1-SC3, and S2-SC19, while other samples showed IC_50_ values ranging from 1 ng/mL to 10.0 μg/mL (Table 1). Additionally, six positive strains, including 1-3, 1-5-22, S1-SC1, S2-SC10, S2-SC16, and S4-SC11, exhibited potent antiplasmodial activity, with IC_50_ values below 1 μg/mL. While the remaining positive strains (5-5-3, S2-SC2, S5-SC5, S5-SC6, and S7-SC9) showed good activity against the K1 strain of *P. falciparum*, with IC_50_ values below 10 μg/mL (Table 1). Not surprisingly, all potent strains were identified as *Streptomyces* species (Table S2). The *Streptomyces* genus is widely known for producing a broad spectrum of bioactive metabolites, as demonstrated by the development of numerous antibiotics and anti-infective drugs currently in use, such as vancomycin, streptomycin, tetracycline, and daptomycin.^12^ However, most of these bacterial strains have been largely isolated from soils. Additionally, many potential actinobacterial strains have been also isolated from extreme environments, such as mangroves and marine habitats worldwide.^13,14^ In Thailand, only a few recent studies have reported antimalarial compounds produced by the *Streptomyces* species isolated from terrestrial and other environments.^4,15^ In this study, we identified six *Streptomyces* isolates, including *S. iranensis*, *S. yogyakartensis*, *S. cacao*, *S. amnesiacs*, *S. phaeoluteichromatogenes*, and *S. albiaxialis* (Tables 1 and 2). These findings suggest that mangrove ecosystems may also serve as a promising source of actinobacteria with antimalarial properties.

**Table 1:** Antimalarial Activity of Selected Thai Actinobacteria^a^

No	Sample	Accession Number	IC_50_ Range (μg/mL)	IC_50_ (μg/mL)
1	1-3	KM678004	6.0 × 10^–4^–0.0094	0.004 ± 0.005*
2	1-5-14	KP339493	3.0 × 10^–4^	3.0 × 10^–4^ ± 0.00*
3	1-5-22	KM678004	0.0046–0.0077	0.006 ± 0.00*
4	5-5-3	KM678024	2.09–3.97	3.03 ± 1.33**
5	S1-SC1	KP339492	0.0052–0.0077	0.006 ± 0.00**
6	S1-SC3	KP339493	3.0 × 10^–4^–8.0 × 10^–4^	5.0 × 10^–4^ ± 0.00**
7	S2-SC2	KM677997	1.81–3.66	2.73 ± 1.31**
8	S2-SC10	KM678000	0.023–0.04	0.03 ± 0.01*
9	S2-SC16	KM678004	0.0034–0.0043	0.004 ± 0.00*
10	S2-SC19	KM678006	4.0 × 10^–4^ – 0.007	4.0 × 10^–4^ ± 0.00*
11	S4-SC11	KP339501	0.14–0.22	0.38 ± 0.04*
12	S5-SC5	KM678021	3.50–3.72	3.61 ± 0.16**
13	S5-SC6	KM678022	3.96–4.66	4.31 ± 0.50**
14	S7-SC9	KM678032	4.42–4.58	4.50 ± 0.12**
15	Artesunate		0.0024–0.0059	0.004 ± 0.00*

a Data are expressed as mean ± SD (*n* = 3) and analyzed using one-way ANOVA followed by Tukey’s post hoc test. *No significant difference, and **p < 0.05 when compared between groups.

**Table 2:**
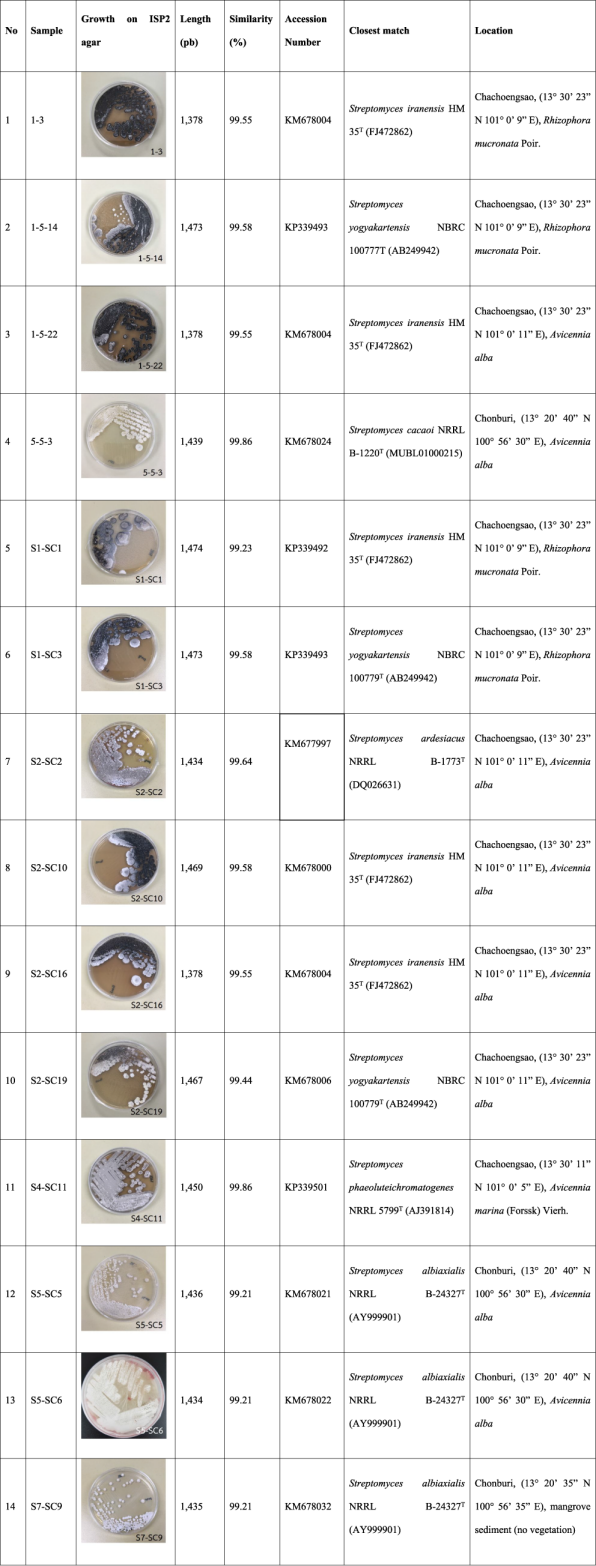
Taxonomic Identification of Selected Actinobacterial Strains Using the 16S rRNA Gene Sequence Analysis

### 2.2 Viral Protein R Inhibitory Activities of the Selected Actinobacterial Extracts

**Figure 2:**
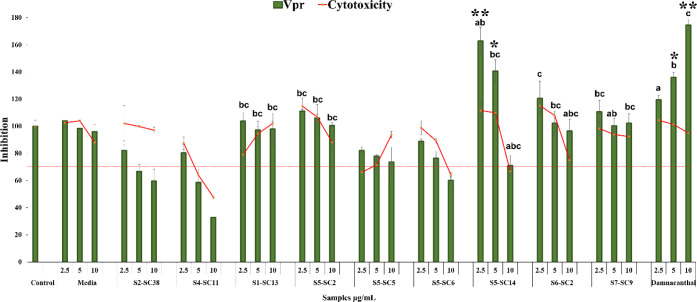
Anti-Vpr activities (green) of the selected Thai actinobacterial isolates and methanolic extracts and their cytotoxicities (red). Data are expressed as mean ± SD (*n* = 3) and analyzed using one-way ANOVA followed by Tukey’s post hoc test. The same letters (a, b, and c) when compared to the positive control damnacanthal (2.5, 5, 10 μg/mL) group indicate significance at *p* < 0.05. And the * and ** indicate significance *p* < 0.05 when compared between groups except among each other. Statistical significance for the samples that showed average to good vpr inhibitory activity was only presented.

Twenty-three actinobacterial isolates were evaluated for their *in vitro* inhibitory effects on the human immunodeficiency virus-1 viral protein R (HIV-1 Vpr) expressed in HeLa cells harboring the TREx plasmid encoding full-length Vpr (TREx-HeLa-Vpr cells). The methanolic extracts (2.5, 5, and 10 μg/mL) were examined for the inhibitory effects on the Vpr activity in the tetracycline-induced Vpr expression in TREx-HeLa-Vpr cells (Figures 2 and S1). The anti-Vpr activity is indicated by the cell proliferation (%) that occurs due to the inhibitory effects of the extracts on the Vpr activity. Damnacanthal (2.5, 5, 10 μg/mL), a potent Vpr inhibitor, was used as a positive control. Blank (CT) represents the TREx-HeLa-Vpr cells grown without any treatment except 1% DMSO. Among the tested methanolic extracts, the extracts of isolates S5-SC14, S6-SC2, S7-SC9, S1-SC13, and S5-SC2 inhibited the Vpr activities in the TREx-HeLa-Vpr cells but were concentration dependent. These isolates displayed a percentage of inhibition equal to or greater than 70% at final concentrations of 2.5, 5.0, and 10.0 μg/mL for each assay. On the other hand, the anti-Vpr activity and cytotoxicity of isolates S2-SC38, S4-SC11, S5-SC5, and S5-SC6 were observed to be moderate, with a smaller percentage of inhibitions meeting the minimum threshold of 70%. These results suggest that while these isolates exhibited some anti-HIV-1 Vpr activity, their effectiveness and safety profiles were not as pronounced as those of the highly potent isolates mentioned earlier. These findings highlight the potential of specific actinomycete isolates to serve as promising candidates for further exploration in developing anti-HIV therapies targeting the Vpr protein. Additionally, they underscore the importance of evaluating potential therapeutic agents’ efficacy and safety profiles, particularly in the context of antiviral drug development. Further investigations into these isolates’ mechanisms of action and molecular targets may provide valuable insights into their therapeutic potential against HIV infection.

### 2.3 16S rRNA Based Identification of Actinobacteria

**Figure 3:**
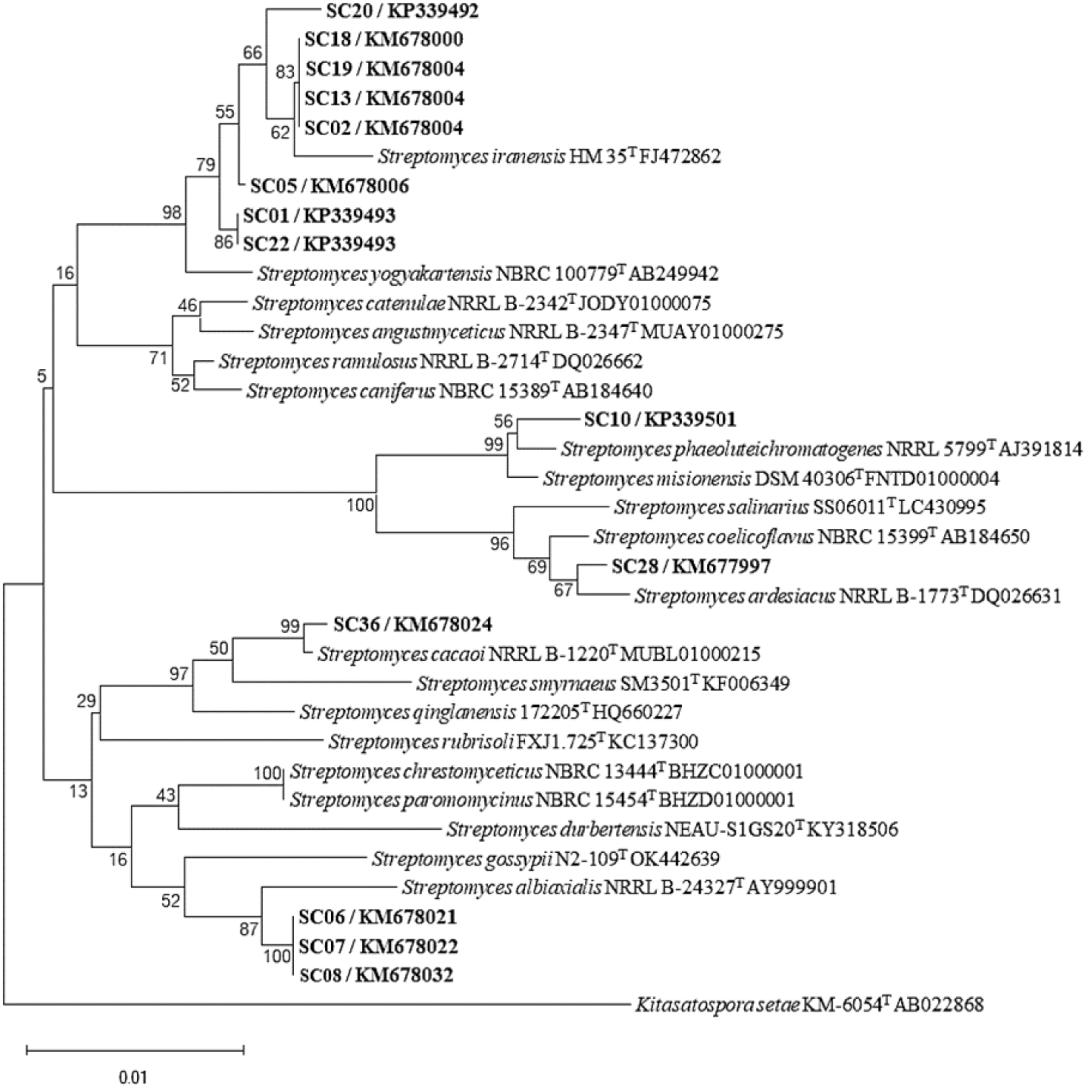
Neighbor joining phylogenetic tree based on almost complete 16S rRNA gene sequences of potent Thai actinobacterial isolates from mangrove sediments demonstrates the relationships with their closely related type strains. Numbers at the nodes indicate levels of bootstrap support based on an analysis of 1,000 resampled data sets. The scale bar indicates 0.01 substitution per nucleotide position.

Genomic DNA of selected actinobacterial isolates was extracted. The yield, purity, and quality were checked by using 0.7% agarose gel and Thermo Scientific NanoDrop One Microvolume UV–vis Spectrophotometers (Thermo Fischer, USA). All selected isolates were identified based on an almost complete 16S rRNA gene sequence analysis using a BLAST search. BLAST results revealed that all selected isolates are members of the genus *Streptomyces*, with the 16S rRNA gene sequence similarity values greater than 99% with their nearest neighbors (Table 2). Specifically, five isolates exhibited the highest sequence similarity to *S. iranensis* HM 35, while three strains showed the highest sequence similarity to *S. yogyakartensis* NBRC 100777. Additionally, three isolates shared the highest sequence similarity with *S. albiaxialis* NRRL entry B-24327. Similarly, the three remaining isolates displayed the highest sequence similarity with *S. cacaoi* NBRC 1220, *S. aiastaticus* NRRL B-1773, and *S. phaeoluteichromatogenes* NRRL 5799. The phylogenetic tree in Figure 3 provides additional context by illustrating the evolutionary relationships among these isolates and their close relatives within the *Streptomyces* genus. This phylogenetic analysis contributed to our understanding of genetic diversity and evolutionary history of these actinobacterial strains, providing insights into their potential ecological roles and biotechnological applications.^16,17^

### 2.4 Global Natural Product Social Molecular Networking Based Metabolite Profiling

**Figure 4:**
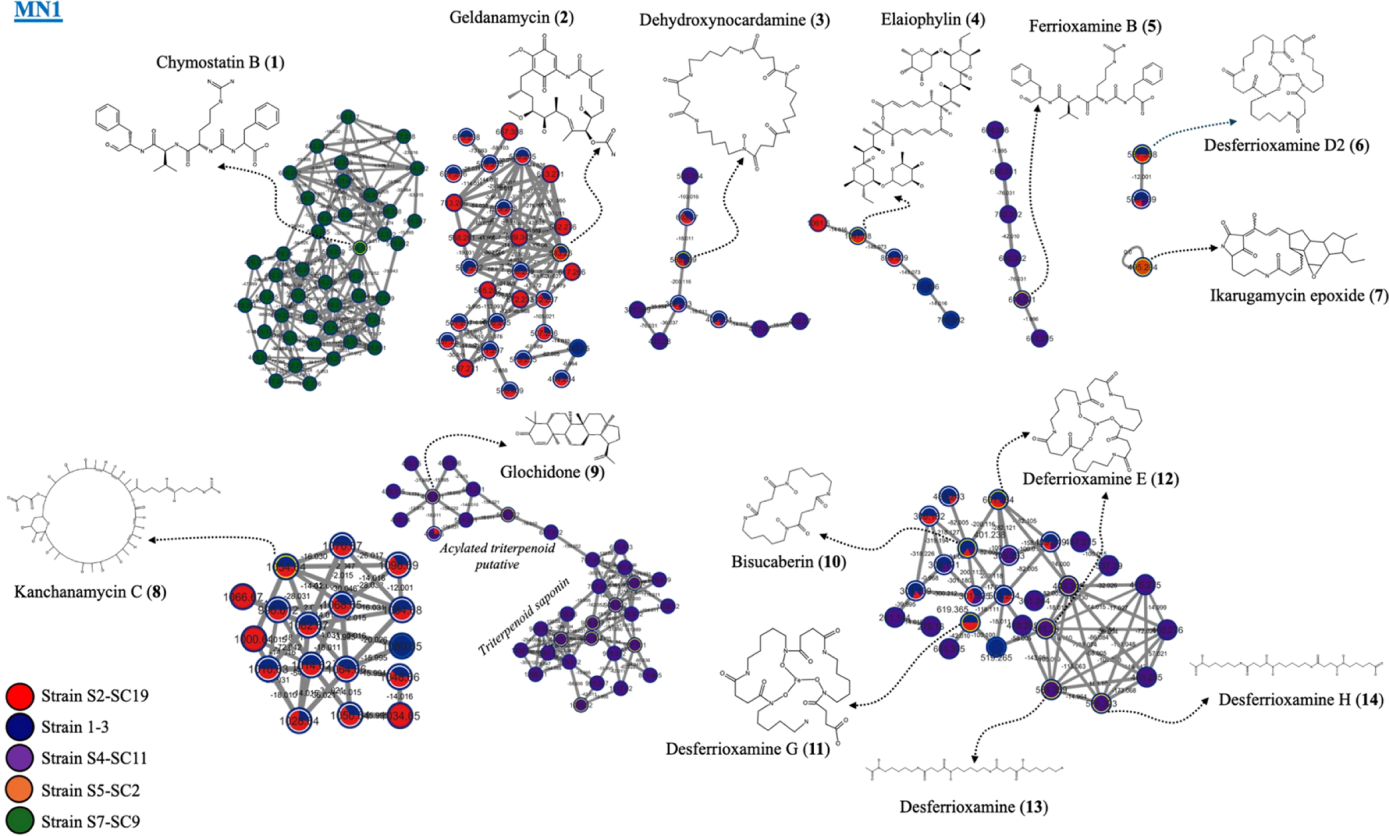
Bioactive actinobacterial molecular network (MN) and clusters of putative bioactive metabolites annotated in positive ion mode by using mass spectrometry. Node colors: red = S2-SC19; blue = 1–3; violet = S4-SC11; orange = S5-SC2; green = S7-SC9.

**Figure 5:**
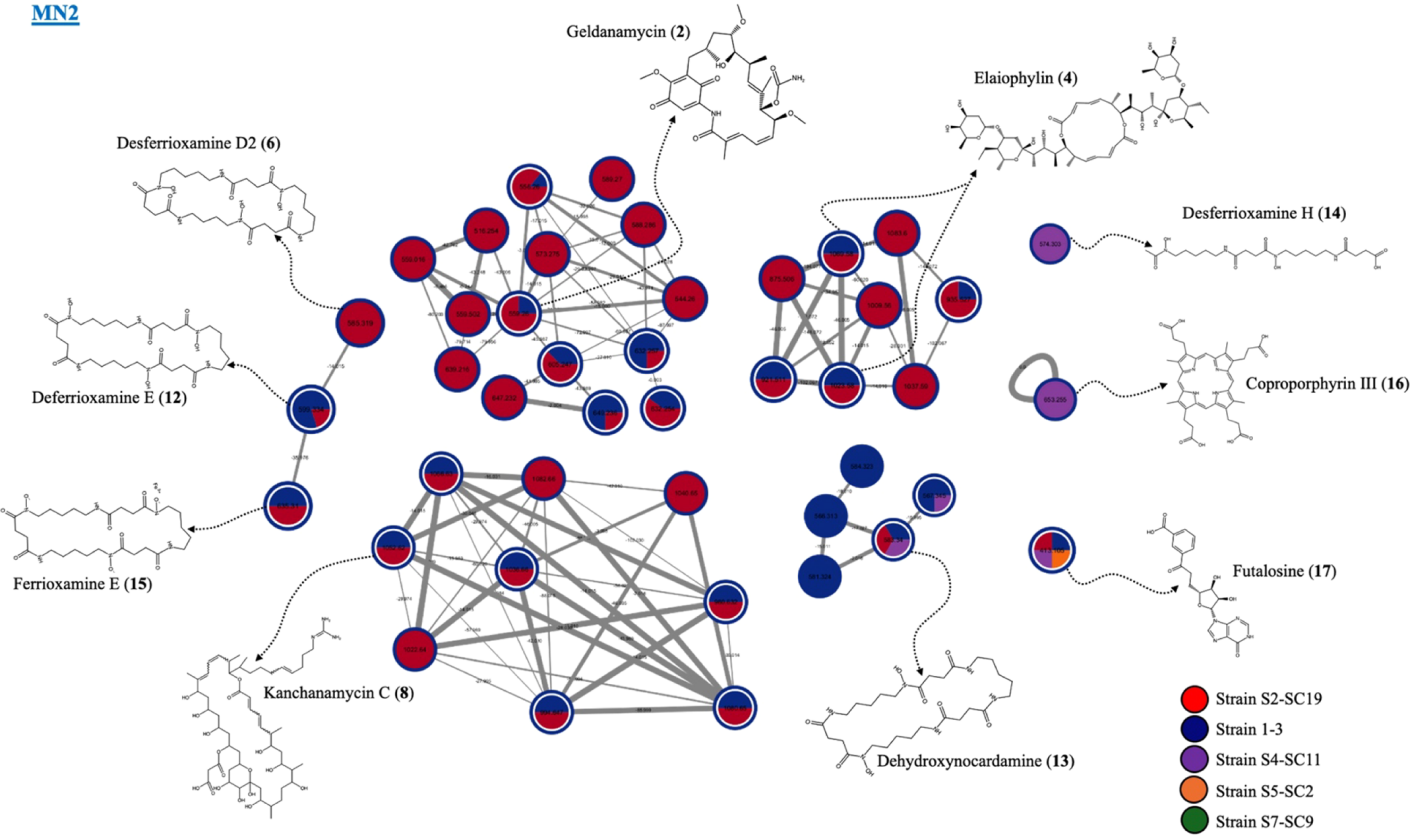
Bioactive actinobacterial molecular network (MN) and clusters of putative bioactive metabolites annotated in negative ion mode by using mass spectrometry. Node colors: red = S2-SC19; blue = 1–3; violet = S4-SC11; orange = S5-SC2; green = S7-SC9.

Based on the antimalarial and anti-Vpr activities of five actinobacterial crude extracts, including 1-3, S4-SC11, S5-SC2, S2-SC19, and S7-SC9, comprehensive and detailed metabolite profiling was conducted using LC-MS/MS and a global natural product social molecular networking (GNPS) based approach. In molecular networking, each spectrum is represented as a node, and nodes are connected by edges when their pairwise similarity exceeds a defined threshold.^11^ Clusters of connected nodes form a molecular family. A total of 3,789 nodes with MS/MS were present in a molecular network (MN), of which 183 hits were annotated (Figure S2). Out of 183 hits, 17 were annotated from the five actinobacterial isolates, with seven hits annotated in both positive and negative MS modes (Figures 4 and 5, Table 3, Table S2 and Figure S2). All annotated compounds were manually confirmed on the StreptomeDB 3.0.^18^ The molecular network is analyzed by representing each compound as a node labeled with its *m*/*z* value. The compounds were categorized into numerous clusters according to the similarity of their MS/MS fragments, which suggests that their fundamental chemical structures are similar.

**Table 3:** Global Natural Product Social Molecular Networking Based Annotated Compounds in Positive and Negative MS/MS Mode Analysis

Annotated compounds	Exact mass	Formula	[M + H]^+^; *m*/*z*	[M-H]^−^; *m*/*z*	Strains	Rt (min)
Chymostatin B (**1**)	593.296	C_30_H_39_N_7_O_6_	[M + H]^+^; 594.302		S7-SC9	7.65
Geldanamycin (**2**)	560.273	C_29_H_40_N_2_O_9_	[M + Na]^+^; 583.263	[M-H]^−^; 559.260	S2-SC19, 1-3	10.10
Dehydroxynocardamine (**3**)	584.715	C_27_H_48_N_6_O_8_	[M + H]^+^; 585.361	[M-H]^−^; 583.340	S2-SC19, 1-3, S4-SC11	5.10
Elaiophylin (**4**)	1025.270	C_54_H_88_O_18_	[M+ Na]^+^; 1047.580	[M-H]^−^; 1023.580	S2-SC19, 1-3	12.20
Ferrioxamine B (**5**)	613.260	C_25_H_48_N_6_O_12_Fe	[M + H]^+^; 614.270		S4-SC11	4.69
Desferrioxamine D2 (**6**)	586.333	C_26_H_46_N_6_O_9_	[M + Na]^+^; 609.322	[M-H]^−^; 585.319	S2-SC19, 1-3	5.92
Ikarugamycin epoxide (**7**)	494.632	C_29_H_38_N_2_O_5_	[M + H]^+^; 495.285		S5-SC2	10.99
Kanchanamycin C (**8**)	1053.630	C_54_H_91_N_3_O_17_	[M + H]^+^; 1054.640	[M-H]^−^; 1052.620	S2-SC19, 1-3	8.66
Glochidone (**9**)	422.697	C_30_H_46_O	[M + H]^+^; 423.362		S4-SC11	9.08
Bisucaberin (**10**)	400.476	C_18_H_32_N_4_O_6_	[M + H]^+^; 401.239		S2-SC19, 1-3, S4-SC11	5.62
Desferrioxamine G (**11**)	618.359	C_27_H_50_N_6_O_10_	[M + H]^+^; 619.366		S2-SC19, 1-3	4.99
Deferrioxamine E (**12**)	600.348	C_27_H_48_N_6_O_9_	[M + Na]^+^; 623.337	[M-H]^−^; 599.334	S2-SC19, 1–3, S4-SC11	6.15
Desferrioxamine (**13**)	560.684	C_25_H_48_N_6_O_8_	[M + H]^+^; 561.361		S4-SC11	5.23
Desferrioxamine H (**14**)	575.317	C_26_H_46_N_6_O_9_	[M + Na]^+^; 576.324		S4-SC11	6.06
Ferrioxamine E (**15**)	653.535	C_27_H_45_FeN_6_O_9_	[M + H]^+^; 654.268	[M-H]^−^; 635.310	S2-SC19, 1-3	6.14
Coproporphyrin III (**16**)	654.720	C_36_H_38_N_4_O_8_		[M-H]^−^; 653.255	S4-SC11	8.63
Futalosine (**17**)	414.374	C_19_H_18_N_4_O_7_		[M-H]^−^; 413.105	S2-SC19, 1-3, S4-SC11, S5-SC2	5.77

The molecular networks MN1 (Figure 4 and Table 3) and MN2 (Figure 5 and Table 3) highlight representative nodes corresponding to structures annotated in GNPS. Significant clusters in both networks were classified into diverse classes of compounds. Among these, **chymostatin B** (**1**; matched 15 MS/MS peaks) was detected in strain S7-SC9, although its activities against malaria and HIV have not been previously reported. **Geldanamycin** (**2**; matched 17 MS/MS peaks) was annotated in strains S2-SC19 and 1-3, and it has been reported to demonstrate antimalarial activity and anti-Hsp90 activity, the latter being a regulator of HIV-1 latency.^19,20^**Dehydroxynocardamine** (**3**; matched 25 MS/MS peaks) was observed in multiple strains (S2-SC19, 1-3, and S4-SC11), with a study reporting its antimalarial activity.^20^**Elaiophylin** (**4**; matched 22 MS/MS peaks) was detected in strains S2-SC19 and has been reported to possess potent antiplasmodial activity.^20^ Siderophores such as **ferrioxamine B** (**5**; matched 19 MS/MS peaks) and **desferrioxamine D2** (**6**; matched 25 MS/MS peaks) were annotated in strains S4-SC11 and S2-SC19, respectively, although their specific bioactivities remain unreported.

Other notable compounds include **ikarugamycin epoxide** (**7**; matched 19 MS/MS peaks) detected in strain S5-SC2, **kanchanamycin C** (**8**; matched 39 MS/MS peaks) identified in strain S2-SC19, and **glochidone** (**9**; matched 15 MS/MS peaks) found in strain S4-SC11. Additional siderophores such as **bisucaberin** (**10**; matched 13 MS/MS peaks), **desferrioxamine G** (**11**; matched 15 MS/MS peaks), **desferrioxamine E** (**12**; matched 26 MS/MS peaks), **desferrioxamine** (**13**; matched 14 MS/MS peaks), and **desferrioxamine H** (**14**; matched 18 MS/MS peaks) were also identified. **Ferrioxamine E** (**15**; matched 7 MS/MS peaks) was detected in strains S2-SC19 and 1-3. Finally, **coproporphyrin III** (**16**; matched 26 MS/MS peaks) and **futalosine** (**17**; matched 8 MS/MS peaks) were found in strains S4-SC11 and across various strains, respectively, although their bioactivities remain unreported. This analysis underscores the metabolic diversity and potential bioactivity of compounds identified in these actinobacterial isolates. It represents a preliminary step toward the discovery of more potent antimalarials and antiretroviral agents.

### 2.5 Isolation of Isolated Antimalarial Compound from Strain S2-SC19

The crude extract of strain S2-SC19 underwent an isolation and purification process, yielding a single compound after the final purification step. The isolated compound (**4**) was isolated as a white, amorphous powder. The molecular formula of **4** was deduced to be C_54_H_88_O_18_ (with 11 degrees of unsaturation) based on a molecular sodium peak at *m*/*z* 1047.5682 [M + Na] (calculated 1047.5863) in the HR-ESI-MS data, suggesting that **4** might be a symmetric compound. The ^1^H NMR spectra of **4** showed the signals corresponding to the six methyl (δ_H_ 0.89–1.6), three methylene (δ_H_ 1.12–1.64), four methine (δ_H_ 1.72–1.94), eight oxygenated methine including one anomeric proton (δ_H_ 5.06), and four olefinic protons (δ_H_ 5.67–6.95) (Table S3 and Figures S3 and S4). The ^1^H NMR data of **4** were similar to those of the elaiophylin (Figure S4), which were isolated from different sources, such as *Streptomyces* sp. strain MCY-846, the rhizosphere of *Paullinia cupana*, and the plant-associated microbes, *Fusarium* wilt of cucumber.^21−23^ Furthermore, to clarify our hypothesis, both isolated compound (**4**) and elaiophylin standard (**4S**), a synthetic product with 85% purity, were injected into the LC-MS and their mass spectra. Subsequently, the MS data of **4** (*m*/*z* 1047.5682 [M + Na]; calculated 1047.5863), were consistent with the MS data of compound **4S** (*m*/*z* 1047.5861 [M + Na]). Moreover, the MS fragmentation peak of **4** in comparison with that of **4S** showed a singlet fragment at 729.38 (Figure S5), which corresponded to the fragmentation of the symmetrical groups bonded to the macrolactone ring of elaiophylin.^22^ By cumulatively comparing the ^1^H NMR and MS spectra, we deduced that the isolated compound is elaiophylin (**4**). However, certain signals exhibited shifts, which might be attributed to differences in analytical methods, instrumentation, or purity of the isolated compound.

The inhibitory activities of **4** and **4S** on the K1 strain of *P. falciparum* and Vero cells were evaluated. Both compounds exhibited strong antimalarial activity, with IC_50_ values of 0.002 and 0.06 μg/mL, respectively (Table 4), while their inhibitory effects on Vero cells were active at 3.98 μg/mL for compound **4** and >4.0 μg/mL for compound **4S**. These results suggest that the isolated compound, which was not fully purified, exhibited stronger activity against malaria parasites, possibly due to the presence of other components within the isolated compound, as observed in the NMR signals. Furthermore, considering the therapeutic efficacy, the results suggest that the isolated compound (**4**) could be a promising candidate for antimalarial drug development. Our findings are consistent with previous studies that indicate potent inhibitory activity of elaiophylin, isolated from *Streptomyces* species, against malaria parasite growth species.^4,24,25^

**Table 4:** Therapeutic Efficacy of Isolated Compound and Its References

Compound	Antimalarial activity against *Plasmodium falciparum* K1 strain (μg/mL)	Cytotoxicity against Vero cells (μg/mL)	Selectivity Index (SI)
Isolated compound (**4**)	0.002 ± 0.002**	3.967 ± 0.144**	1803.114
elaiophylin standard (**4S**)	0.060 ± 0.010*	>4.000^a^	ND
Artesunate	0.040 ± 0.011**	ND	ND
Atovaquone	0.062 ± 0.014*	ND	ND
Doxorubicin	ND	15.189 ± 0.414**	ND

a Data are expressed as mean ± SD (n = 3) and analyzed using one-way ANOVA followed by Tukey’s post hoc test. ^b^ Highest tested concentration * No significant difference, and **p < 0.05 when compared between groups.

### 2.6 Genome Analysis of Actinobacteria Strain S2-SC19

*Streptomyces* sp. S2-SC19 was isolated from mangrove sediment associated with *Avicennia alba* in Chachoengsao Province, Thailand. Strain S2-SC19 developed a well-defined substrate mycelium and an aerial mycelium featuring long, flat spore chains with rugose ornamentation (Figure S6). On certain media, these chains coalesced into dark spore masses as they matured.

Sequencing was performed using a paired-end approach, generating a total of 3.349 million reads and 503.239 million bases (Table S4). A small fraction of the reads (1.478% or 51,911 reads) and a more substantial proportion of bases (21.558% or 113.76 million bases) were filtered out during quality control. The estimated sequence error rate was 0.06%, indicating high data accuracy. Additionally, 874,165 reads and 57.974 million bases underwent adapter trimming. After QC, an automatic tool for filtering, trimming, error removal, and overall quality control of FASTQ sequencing data was used for quality assessment (Table S4).

The complete linear genome of strain S2-SC19 is 10,585,008 bp in length with a GC content of 71.44%. It comprises 237 contigs with an N50 value of 88,717. The chromosome contains 8,869 genes, including 8,775 protein-coding genes, 6 rRNA genes, 87 tRNA genes, and 1 tmRNA gene. Seventeen genomes of *Streptomyces* strains, including *Streptomyces hygroscopicus* NBRC 13472, *Streptomyces asiaticus* DSM 41761, and *Streptomyces iranensis* DSM 41954, were selected for comparative genomics analysis with strain S2-SC19 using the Type-Strain Genome Server.^26^ As a result, strain S2-SC19 was found to be evolutionarily closest to *S. asiaticus* DSM entry 41761. Orthologous gene cluster analysis showed that the two strains share 3,724 orthologous clusters out of 3,742 clusters in strain S2-SC19 and 3,776 clusters in *S. asiaticus* DSM 41761.

Prediction of biosynthetic gene clusters (BGCs) and secondary metabolites was performed using antiSMASH 7.0.^27^ A total of 103 putative BGCs were identified in strain S2-SC19, including 37 polyketides, 25 nonribosomal peptides, 4 terpenes, 2 ribosomally synthesized and post-translationally modified peptides (RiPPs), and 9 other secondary metabolites (Table S5). Among these, 10 putative BGCs displayed high similarity (>70% gene similarity) to known clusters for compounds such as 2-methylisoborneol, pristinol, ochronotic pigment, legonoxamine A/legonosamine B, echoside, ε-poly-l-lysine, ectoine, feglymycin, tetrafibricin, and rhizomide. Additionally, 27 putative BGCs showed moderate similarity (30%–70% gene similarity) to known BGCs, 41 showed low similarity (<30% gene similarity), and 25 had no similarity to any known gene clusters. The presence of these cryptic BGCs suggests that strain S2-SC19 may be a valuable source of novel bioactive compounds.

**Figure 6:**
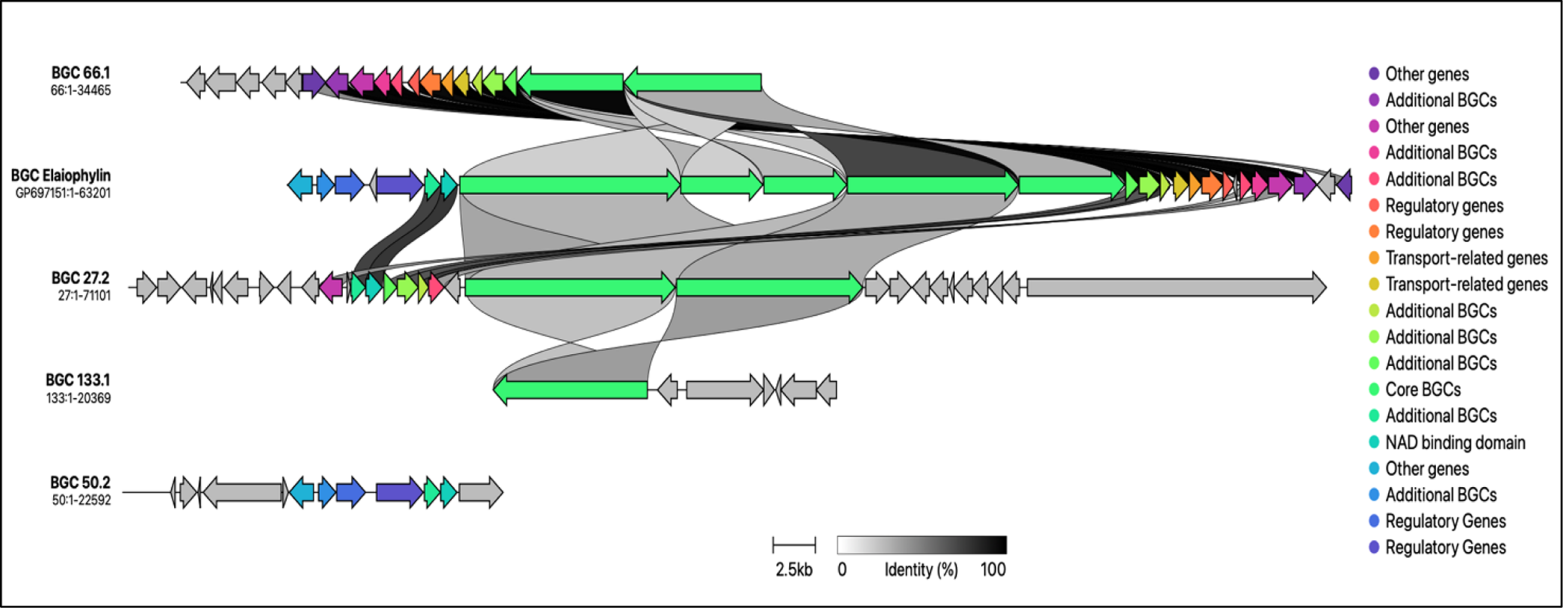
Alignment of the elaiophylin BGC from the MIBiG database with the predicted elaiophylin-like BGCs found in regions 27.2, 50.2, 66.1, and 133.1 of *Actinobacteria* strain S2-SC19. Genes are color-coded based on their functional groups, and homologous genes are connected by shaded regions that indicate the percentage of amino acid identity between their protein products.

**Figure 7:**
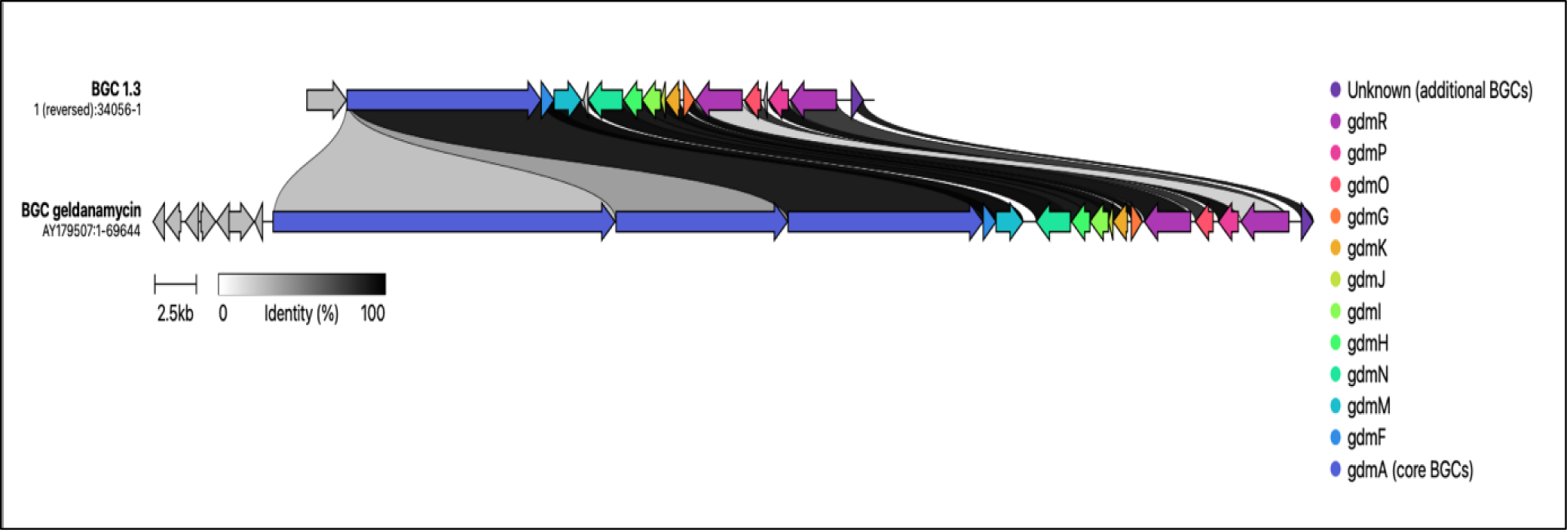
Alignment of the geldanamycin BGC from the MIBiG database with the predicted geldanamycin-like BGCs found in region 1.3 of *Actinobacteria* strain S2-SC19. Genes are color-coded based on their functional groups, and homologous genes are connected by shaded regions that indicate the percentage of amino acid identity between their protein products.

The antiSMASH analysis revealed the presence of BGCs responsible for the production of elaiophylin (also known as azalomycin and efomycin) and geldanamycin. Previously, elaiophylin and geldanamycin BGCs were reported together in several sequenced genomes of *Streptomyces* sp.^28^ Yin et al.^29^ stated that elaiophylin and geldanamycin are usually coproduced and suggested that these two biosynthetic pathways might be intriguingly linked, as both use malonyl-CoA and methyl malonyl-CoA as extension units. In the assembly, BGC 66.1 exhibited the highest gene similarity at 66%, followed by BGCs 27.2, 133.1, and 50.2, with gene similarities of 45%, 39%, and 37%, respectively, to BGCs associated with elaiophylin-like compounds. This similarity degree was verified using the CAGECAT tool^30^ (Figure 6 and Table S6). Subsequently, BLASTP analysis of the genome sequence of strain S2-SC19 revealed a gene, *ElaA*, which shares significant sequence similarity (>98%) with the Ela1 module, previously identified in *Streptomyces* sp. GMR22,^29^ and involved in elaiophylin biosynthesis. These findings suggest that strain S2-SC19 possesses the genetic machinery required for the production of elaiophylin and its derivatives. Meanwhile, BGC 1.3 showed the highest gene similarity at 69% to that of a BGC associated with geldanamycin. This was confirmed by the CAGECAT tool (Figure 7).^30^ Similarly, the geldanamycin BGC in strain S2-SC19 exhibited high similarity to that in *Streptomyces* sp. GMR22. Further investigation into the elaiophylin biosynthetic pathway and its potential links with the geldanamycin biosynthetic pathway could facilitate the manipulation of elaiophylin production and its derivative compounds for bioactive applications.

## 3 Conclusions

A total of 164 actinobacteria were isolated and characterized from mangrove samples collected in the eastern region of Thailand. These isolates were identified as members of the *Streptomyces* species. Among them, more than ten demonstrated significant inhibitory activity against the chloroquine-resistant strain of *P. falciparum*. These isolates showed the highest similarity to *S. iranensis*, *S. yogyakartensis*, *S. cacaoi*, *S. ardesiacus*, *S. phaeoluteichromatogenes*, and *S. albiaxialis*. Additionally, few of them closely related to *S. albiaxialis* exhibited potent activity against TREx-HeLa-Vpr cells. MS/MS-guided molecular networking analysis highlighted the metabolic complexity of the isolates, revealing a diverse array of structurally distinct compounds. These included chymostatin B (**1**), geldanamycin (**2**), dehydroxynocardamine (**3**), elaiophylin (**4**), ikarugamycin epoxide (**7**), kanchanamycin C (**8**), glochidone (**9**), bisucaberin (**10**), coproporphyrin III (**16**), and futalosine (**17**). Additionally, multiple siderophores were detected, including ferrioxamine B (**5**), desferrioxamine D2 (**6**), desferrioxamine G (**11**), desferrioxamine E (**12**), desferrioxamine (**13**), desferrioxamine H (**14**), and ferrioxamine E (**15**). Notably, a known macrolide compound (**4**) was isolated from actinobacteria strain S2-SC19 using molecular networking and bioassay-guided fractionation approaches. The isolated compound shared a high similarity to elaiophylin according to GNPS molecular networking, comparative LCMS analysis with standard, ^1^H NMR, and genome analysis. Moreover, the genomic-based taxonomic identification revealed the strain S2-SC19 as *Streptomyces asiaticus*, and to the best of our knowledge, it is a new source of elaiophylin. Remarkably, isolated compound (**4**) exhibited potent antimalarial activity against the *Plasmodium falciparum* K1 strain, with an exceptional selectivity index (SI) of 1,803.1, indicating a wide therapeutic safety margin. Our study suggests that the strain S2-SC19 may be a promising alternative source for up-scale production and targeted isolation of antiplasmodial compounds, which are confirmed by spectroscopic approach and biosynthetic gene clusters.

## 4 Experimental Section

### 4.1 Chemicals and Reagents

Roswell Park Memorial Institute (RPMI) 1640 cell culture medium (Invitrogen, USA), HEPES (Himedia, India), sodium bicarbonate (Sigma Life Science, USA), hypoxanthine (Himedia, India), gentamicin (Invitrogen, USA), AlbuMAX II (Invitrogen, USA), DMSO (RCI Labscan, Thailand), artesunate (Sigma-Aldrich, USA), 3-acetylpyridine adenine dinucleotide (APAD) (Sigma-Aldrich, USA), Triton X-100 (Loba, India), TRIS buffer (Sigma Life Science, USA), nitroblue tetrazolium (Himedia, India), phenazine ethosulfate solution (Himedia, India), Dulbecco’s modified Eagle’s medium (DMEM) (Invitrogen, USA), penicillin/streptomycin solution (Invitrogen, USA), fetal bovine serum (FBS) (Sigma-Aldrich, USA), 3-(4,5-dimethythiazol-2-yl)-2,5-diphenyl tetrazolium bromide (MTT) (Invitrogen, USA), trypsin solution (Invitrogen, USA), ethyl alcohol (AR grade, >99%, SM Chemicals, Thailand), methanol (AR grade, >99%, RCI Labscan, Thailand), methanol (MeOH) (HPLC grade, >99%, RCI Labscan, Thailand), and acetonitrile (ACN) (HPLC grade, >99%, RCI Labscan, Thailand) were used.

### 4.2 Collection of Thai Actinobacteria

A collection of actinobacteria was isolated from mangrove soils collected in the eastern region of Thailand. Mangrove sediments were treated and cultured on standard culture media as previously described.^3,31,32^ Purified colonies were grown on ISP2 agar medium (4.0 g/L yeast extract, 4.0 g/L glucose, 10.0 g/L malt extract, and 15 g/L agar powder) and cultured at 28 °C for 2 weeks.

### 4.3 Preparation of Crude Extracts

Agar cultures of each actinobacterial isolate were cut into pieces and extracted by maceration in AR-grade methanol (RCI Labscan, Thailand). The extraction was carried out overnight in a shaker incubator. After being filtered using filter papers, the solid materials (agar residue) were discarded, and the extracts were concentrated using a rotary evaporator to yield crude extracts. The crude extracts were stored at −20 °C until LCMS analysis.

### 4.4 *In Vitro* Antimalarial Activity of Extracts

A parasite Lactate Dehydrogenase (pLDH) assay with modifications^33^ was performed to assess the inhibitory activity of the crude extracts of 164 actinobacterial isolates. The chloroquine-resistant K1 strain of *P. falciparum* was maintained in RPMI 1640 culture medium (Invitrogen, USA). In brief, the complete malaria culture medium (cMCM) was supplemented with 4.7 g/L HEPES (Himedia, India), 2 g/L NaHCO_3_, 0.1 g/L hypoxanthine (Himedia, India), 25 mg/mL gentamicin (Invitrogen, USA), and 0.5% Albumin Bovine Serum (Invitrogen, USA). The *P. falciparum* K1 strain was grown using noninfected type B+ red blood cells (RBCs), maintained at 2% hematocrit, and incubated in a CO_2_ incubator at 37 °C. According to a previous publication,^33^ parasite-infected RBCs were mixed with cMCM and dispensed into 96-well plates (2% hematocrit, 2% parasitemia). Crude extracts of actinobacteria were prepared in DMSO (RCI Labscan, Thailand) to obtain 100 and 10 mg/mL stock solutions for the primary screening and the determination of IC_50_ values, respectively. 2-fold dilutions were prepared to obtain final extract concentrations ranging from 10 to 0.31 mg/mL. Crude extracts (1 μL) were added to each well. Artesunate was used as a positive control, and DMSO was used as a negative control. In all samples, negative and positive controls were maintained at 0.5% for DMSO concentration. The culture was incubated at 37 °C with high relative humidity (95%) and a stable CO_2_ level (5%) for 72 h. After 72 h of incubation, the tested plates were subjected to cycles of freezing for 30 min at −20 °C and thawing for 30 min at 37 °C (three times). When the infected RBCs were completely lysed, 20 μL of the culture was transferred to new 96-well plates containing 100 μL of Malstat reagent, and the plates were incubated at room temperature for 30 min. Next, 20 μL of NBT/PES solution was added to each well, and the plates were incubated in the dark for 30 min. The absorbance was measured at 650 nm by using a microplate reader. Parasite growth inhibition was calculated using the following equation: inhibition (%) = [100 – (abs of treat well-abs of noninfected well)/(abs of nontreated well-abs noninfected well)] × 100. The inhibition percentages were used to plot a nonlinear dose–response curve based on the concentrations. The half-maximal inhibitory concentration (IC_50_) value of the crude extracts was determined from the latter.

### 4.5 *In Vitro* Vpr Inhibitory Activities and Cytotoxicity

Inhibition of HIV-1 viral protein R (Vpr) activity by actinobacterial crude extracts was performed against Vpr-induced TREx-HeLa cells with some modifications.^10^ To summarize, the TREx-HeLa-Vpr cells were maintained in minimum essential medium (αMEM), supplemented with 10% fetal bovine serum (Sigma-Aldrich, Germany), 1% penicillin (10 mg/mL)-streptomycin (10 mg/mL) (Gibco, USA), 0.005 mg/mL blasticidin, and 0.05 mg/mL zeocin. A 48-well plate containing 150 μL of complete culture medium was prepared. The TREx-HeLa-Vpr cells were then dispensed into each well, and the plates were incubated in 95% humidified air with 5% CO_2_ at 37 °C for 24 h. After the cells were incubated for a day, they were exposed to crude extract solution, with the final concentration in the wells ranging from 2.5 to 10 μg/mL, and incubated for 48 h. After incubation, 50 μL of 10% MTT solution was added to the plates and incubated for 3 h. Next, 200 μL of DMSO was added after the culture medium was discarded from each well. The plates were incubated for 10–15 min, and Vpr activity was measured at 570 nm using a Multiskan SkyHigh Microplate Reader (Thermo Fisher Scientific, USA). The percentage of cell viability (%) was calculated using the following formula: cell viability (%) = [(Absorbance of treated cells − absorbance of blank)/(absorbance of untreated cells − absorbance of blank)] × 100. The cytotoxicity of the crude extracts was assessed by using the same MTT assay without the induction of tetracycline. The TREx-HeLa-Vpr cells with 1% DMSO were used as a negative control or blank (CT) representing the cell viability and growth. Damnacanthal was used as a positive control. The sample media represent the media blank used for actinobacterial culture.

### 4.6 Genomic DNA Extraction and Taxonomic Identification

Actinobacteria that exhibited toxic effects against the K1 strain of *P. falciparum* and TREx-HeLa-Vpr cells were selected for taxonomic identification. A liquid culture of each strain was required to perform genomic DNA extraction, followed by a GenElute Bacterial Genomic DNA kit (Sigma-Aldrich, Germany) with some modifications. Briefly, a 3-day bacterial culture was harvested in a 1.5 mL Eppendorf by centrifuging at 15,000 × *g* for 2 min before discarding the culture media. Cells were resuspended in 200 μL of lysozyme solution and incubated 1 h at 37 °C. Then, 20 μL of proteinase K solution was added to the sample, followed by 200 μL of lysis solution C. Samples were mixed and incubated for 30 min at 55 °C. At the same time, binding columns were placed into a 2 mL collection tube. The binding columns were prepared by adding 500 μL of column preparation solution and centrifuged at 12,000 × *g* for 1 min. The flow-through liquid was discarded. 200 μL of absolute ethanol was added to the lysates of the previous step and mixed thoroughly. The homogeneous contents were transferred into the prepared binding columns and centrifuged at 9,000 × *g* for 1 min. The flow-through liquid was discarded, and new 2 mL collection tubes were replaced. The contents were washed with 500 μL of wash solution 1 and centrifuged at 9,000 × *g* for 1 min. The flow-through liquid was discarded before washing a second time with 500 μL of wash solution concentrate and centrifuge. 100 μL of elution was added onto the center of the column and incubated for 5 min before centrifuging (9,000 × *g* for 1 min). The library construction was prepared using the S-1.2x-TN5 library PCR Mix Kit (Tsingke, China). The partial 16S rRNA gene sequencing using universal primers for bacteria, 27F (5′-AGAGTTTGATCCTGGCTCAG-3′) and 1492R (5′-TACGGCTACCTTGTTACGACTT-3′), was performed on an Illumina HiSeq sequencing platform, using MiSeq Reagent Micro Kit V2 designed for the MiSeq system (Illumina, USA). The forward and reverse sequences were aligned, and consensus sequences were obtained using Unipro UGENE software.^34^ The sequences were compared to those reference sequences present in EzBioCloud by using the basic local alignment search tool (BLAST). The sequences of selected isolates were then aligned, and a phylogenetic tree was constructed and edited using MEGA version 11.^35^

### 4.7 Liquid-Chromatography and Molecular Networking Analysis

Samples ranging from 0.03 to 100 mg were dissolved in 75% methanol and adjusted to 50 to 300 mg/L concentrations. After centrifugation at 12,000 × *g*, 25 °C for 15 min, the resulting supernatant was carefully transferred and filtered through a 0.22 μm Hydrophilic PTFE membrane. Next, mass identification was carried out using ultrahigh-performance liquid chromatography-high-resolution mass spectrometry (UHPLC/HRMS/MS) with an orbitrap mass analyzer (Thermo Fisher Scientific, USA). Using a Hypersil GOLD Vanquish C18 column (2.1 × 100 mm, 1.9 μm, Thermo Scientific) with a guard column, separation occurred at 40 °C with a flow rate of 0.4 mL/min. Mobile phases A and B consisted of 0.1% formic acid (FA) in water and acetonitrile, respectively. The elution process commenced with 5% B for 4 min, followed by a linear increase to 90% B over 10 min. Subsequently, the column was flushed with 90% B for 4 min. Then, the concentration of phase B decreased to 5% over 1 min before returning to the initial conditions, resulting in a total runtime of 25 min. The column was re-equilibrated before subsequent analyses. For MS data acquisition, heated electrospray ionization (HESI) was employed with a spray voltage of 3.5 kV for positive mode and 2.5 kV for negative mode. Gas settings were as follows: sheath gas, auxiliary gas, and sweep gas at 45 AU, with flow rates of 10 and 2 AU, respectively. The capillary temperature was maintained at 320 °C. Full scan MS and data-dependent analysis MS were performed at resolutions of 120,000 and 30,000, respectively, covering a scan range of 100–1500 *m*/*z*. Raw data were acquired and processed using Compound Discoverer software v 3.3.

The LC/MS data were further converted using Compound Discoverer software 3.3 (Thermo Fisher Scientific, USA) to mzXML before further processing. The mass spectral data were searched in the spectral library and molecular networking using the GNPS platform^11^ for the possible annotation of known compounds and derivatives. The precursor and fragment ion mass tolerances were set at 0.05 Da. The advanced network parameters were set as follows: minimum pair cosine, 0.7; network topK, 10; maximum connected component size, 100; minimum matched fragment ions, 6; and minimum cluster size, 2. Advanced library search and minimum matching of the library search were set at 6, with a threshold score of 0.7. The default values were used for the other parameters. After the molecular library search, the data obtained were used to visualize molecular networks using Cytoscape, version 3.10.^36^ All MS data are publicly available in MassIVE (https://massive.ucsd.edu) under the accession numbers MSV000096220 and MSV000096221.

### 4.8 Bioassay-Guided Antimalarial Compound Isolation from Strain S2-SC19

The strain S2-SC19 of actinobacteria was grown on ISP2 agar medium for 21 days at 28 °C. The culture agar was cut into pieces and extracted in methanol on a rotary shaker overnight. The methanolic extract (14.0 g) was fractionated by a Sephadex LH-20-packed column using methanol (MeOH). MeOH fractions were collected at a flow rate of 3 mL/min. The MeOH fractions were further purified by preparative HPLC using a step gradient of ACN-H_2_O (from 5:95 to 80:20 for 50 min to yield isolated compound (*t*_R_ = 25.15 min, 2.0 mg).

#### 4.8.1 NMR Analysis

1D (^1^H) NMR spectra were acquired in deuterated methanol (MeOD) using a BRUKER Ascend 500 NMR spectrometer equipped with a CryoProbe Prodigy.

#### 4.8.2 Assessment of Antimalarial Activity

*In vitro* antimalarial assay was performed as previously described.^33^ The chloroquine-resistant K1 strain of *P. falciparum* was routinely cultured in RPMI 1640 medium (Invitrogen, USA) containing 0.5% AlbuMAX II (Invitrogen, USA), 4.7 g/L HEPES (Himedia, India), 2 g/L NaHCO_3_, 0.1 g/L hypoxanthine (Himedia, India), and 25 mg/mL gentamicin (Invitrogen, USA) at 37 °C in a 5% CO_2_ incubator. A pLDH assay was performed to determine the parasite growth inhibitory activity. For antimalarial activity, the K1 strain of *P. falciparum* culture was dispensed in 96-well plates (2% hematocrit, 2% parasitemia). The parasites were treated with various concentrations of the isolated compound (**4**) and elaiophylin standard (**4S)** (Toronto Research Chemicals, Canada lot no. 2023-MFI-001; 85% purity) for 72 h. The tested plates were frozen 1 h (−80 °C) and thawed 1 h (37 °C). In new 96-well plates, 20 μL of lysed parasite culture was mixed with Malstat reagent (100 μL) and NBT/PES solution (20 μL) before being incubated in the dark for 1 h. Activity was measured using a Multiskan SkyHigh microplate reader (Thermo Fisher Scientific, USA) at 650 nm. DMSO was used to normalize parasite growth. Artesunate and atovaquone were used as positive antimalarial drug controls.

#### 4.8.3 Assessment of Cytotoxic Effects

Kidney epithelial cells of an African green monkey (Vero cells) were maintained in DMEM medium (Invitrogen, USA) supplemented with 10% FBS (Invitrogen, USA) and 1% penicillin (10 mg/mL)-streptomycin (10 mg/mL) (Invitrogen, USA) at 37 °C in a CO_2_ incubator. Toxic effects of **4** and **4S** were carried out using the MTT assay.^33^ 10^4^ Vero cells/mL were incubated in 96-microplates for 24 h. The **4** and **4S** were serially diluted to obtain final concentrations (4.00–0.001 μg/mL). DMSO was used to normalize cell viability. Tested plates were incubated for 24 h at 37 °C in 95% relative humidity and 5% CO_2_. After 24 h of incubation, 50 μL of MTT solution was added to each well, and the plates were incubated for 3 h. Then, MTT solution was discarded before adding DMSO (100 μL) to dissolve formazan crystals. Cell viability was determined at 560 and 670 nm of UV wavelengths using a Multiskan SkyHigh microplate reader (Thermo Fisher Scientific, USA). Cytotoxicity was determined using a nonlinear regression curve. The efficacy of **4** and **4S** was determined using the following equation: selectivity index (SI) = cytotoxicity concentration (CC_50_)/antimalarial activity (IC_50_).

### 4.9 Genome Analysis of Actinobacteria Strain S2-SC19

DNA extraction of the strain S2-SC19 was carried out as previously described in 4.6. The genome sequencing of Actinobacteria strain S2-SC19 was done by BIONICS Co., Ltd. (Republic of Korea). Briefly, the library construction was prepared using the Illumina TruSeq Nano DNA Sample Prep Kits (Illumina, Inc., USA). The sequencing was conducted using the Illumina platform with the Hiseq machine, utilizing the Miseq reagent kit V3 300 cycles (Illumina, Inc., USA). For a single sample processed offline, the data volume reached 1 GB per sample when at least 90% of the committed data volume was utilized. The raw data were processed to remove splices, contaminated sequences, and low-quality reads. Then, data outputs and data quality control were done and analyzed by a bioinformatician from U2Bio Co., Ltd. (Thailand). This whole genome shotgun project has been deposited at DDBJ/ENA/GenBank under accession JBHEQJ000000000.1. The data have been deposited with links to BioProject accession number PRJNA1158514 in the NCBI BioProject database (https://www.ncbi.nlm.nih.gov/bioproject/).

### 4.10 Data Analysis

Experiments were conducted independently in triplicate and were repeated three times. Data are expressed as the mean ± the standard deviation. The CC_50_ and IC_50_ values were calculated from drug treatment dose–response curves using nonlinear regression analysis in GraphPad Prism (GraphPad Software, Inc., CA). Statistical analysis was performed using ANOVA followed by Tukey’s post hoc test for pairwise comparisons. Significance levels were defined as *p* ≤ 0.05.

## Supporting Information Available

Supplementary antimalarial activity of 164 actinobacterial extracts, GNPS molecular networks and annotated compounds details, ^1^H NMR and LCMS spectra of **4** and **4S**, AntiSMASH results of strain S2-SC19, and scanning electron micrographs of the strain S2-SC19 (PDF)
