# Supplementary material for: Nontargeted Metabolomics of Streptomyces Sourced from Thailand Reveals the Presence of Bioactive Metabolites
Source: ACS Omega. 2025 Mar 11;10(11):11567–79. doi: 10.1021/acsomega.5c00669 (PMC11947806; doi:10.1021/acsomega.5c00669)
Supplement: Supplementary file 1 — ao5c00669_si_001.pdf [file ao5c00669_si_001.pdf]

Supplementary File

Nontargeted Metabolomics of *Streptomyces* sourced from Thailand Reveals the Presence of Bioactive Metabolites

Yuwathida Sunghanghwa[a], Atchara Paemanee[b], Kiep Minh Do[c], Mathurin Meethangdee[d], Michaela Plechatá[e,f], Wasu Pathom-aree[d], Chuchard Punsawad[g], Hiroyuki Morita[h], Sithichoke Tangphatsornruang[b], Zdenek Kamenik[f], Amit Jaisi[i,j] *

Institution Adress:

[a]College of Graduate Studies, Walailak University, Thasala, Nakhon Si Thammarat 80160, Thailand

[b]National Center for Genetic Engineering and Biotechnology, National Science and Technology Development Agency, Pathum Thani 12120, Thailand

[c]Faculty of Pharmacy, Nam Can Tho University, Can Tho 900000, Vietnam

[d]Department of Biology, Faculty of Science, Chiang Mai University, Chiang Mai 50200, Thailand

[e]University of Chemistry and Technology Prague, Technická 5, 160 00 Prague 6-Dejvice, Czech Republic

[f]Institute of Microbiology of the Czech Academy of Sciences, Czech Republic

[g]School of Medicine, Walailak University, Thasala, Nakhon Si Thammarat 80160, Thailand

[h]Institute of Natural Medicine, University of Toyama, Toyama 930-0194, Japan

[i]School of Pharmacy, Walailak University, Thasala, Nakhon Si Thammarat 80160, Thailand

[j]Biomass and Oil Palm Center of Excellence, Walailak University, Thasala, Nakhon Si Thammarat 80160, Thailand

Correspondence

amit.ja@mail.wu.ac.th

Table S1 Antiplasmodial activity of Thai actinobacteria

No	Sample code	WU code	% Inhibition at 0.5 mg/ml	Antimalarial activity
1	1-3	WU02	100.38 ± 0.35	+++
2	1-5-14	WU01	72.89 ± 4.55	++
3	1-5-22	WU13	75.14 ± 3.50	++
4	1-17		72.49 ± 9.54	++
5	2		37.22 ± 2.82	-
6	2-2	WU25	47.17 ± 1.96	-
7	2-3		62.43 ± 3.15	+
8	2-4-9		5.54 ± 5.78	-
9	2-7		31.06 ± 3.18	-
10	2-8		42.72 ± 1.40	-
11	2-14		28.67 ± 2.42	-
12	2-18		60.42 ± 1.35	+
13	2-21		37.69 ± 1.58	-
14	2-24		52.67 ± 5.28	+
15	2-25		58.10 ± 1.90	+
16	2-27	WU03	39.68 ± 2.11	-
17	2-30	WU24	37.69 ± 0.30	-
18	2-32		43.79 ± 2.69	-
19	2-33		61.62 ± 1.15	+
20	2-34		7.52 ± 5.85	-
21	2-39		73.35 ± 1.20	++
22	2-42		9.18 ± 13.24	-

No	Sample code	WU code	% Inhibition at 0.5 mg/ml	Antimalarial activity
23	2-53		48.03 ± 2.83	-
24	4-3-6		52.47 ± 3.81	+
25	4-3-7		39.01 ± 2.34	-
26	5-1-1		34.57 ± 3.84	-
27	5-3-3		72.49 ± 1.18	++
28	5-5-3	WU36	51.94 ± 1.11	+
29	5-18		52.87 ± 1.78	+
30	7-1-3		52.13 ± 2.99	+
31	7-1-4	WU26	23.50 ± 7.30	-
32	7-1-7		33.56 ± 5.01	-
33	7-1-10		51.24 ± 5.01	+
34	7-1-13		30.70 ± 4.90	-
35	10	WU31	84.70 ± 2.02	+++
36	14		62.19 ± 3.45	+
37	A5		19.82 ± 11.25	-
38	AV2-1-5		62.39 ± 1.50	+
39	AV2-1-9		59.88 ± 2.53	+
40	AV2-1-13		21.39 ± 2.93	-
41	AV2-1-26		72.93 ± 0.26	++
42	AV2-1-28		19.55 ± 3.19	-
43	AV2-4-7		41.31 ± 3.98	-
44	AV2-4-10	WU29	19.35 ± 4.25	-
45	AV2-5-14	WU16	11.19 ± 7.17	-

No	Sample code	WU code	% Inhibition at 0.5 mg/ml	Antimalarial activity
46	AV2-5-15	WU15	34.38 ± 4.09	-
47	AV2-6-3		35.26 ± 1.90	-
48	AV2-7-5		34.44 ± 8.42	-
49	AV2-49		72.46 ± 1.24	++
50	AV5-2-13		36.48 ± 2.24	-
51	AV5-5-3		75.18 ± 4.21	++
52	R1-1A/A101		13.77 ± 5.95	-
53	R1-1A/B119		66.85 ± 1.30	+
54	R1-2A/B102		70.62 ± 5.64	++
55	R1-2A/N20 AO		64.30 ± 1.74	+
56	R1-2A/O104		-	-
57	R1-2B/A502	WU30	2.77 ± 3.26	-
58	R1-2B/D103		37.85 ± 1.40	-
59	R1-2B/D803		37.05 ± 5.39	-
60	R1-2B/D805		35.93 ± 4.26	-
61	R1-2B/H206		25.23 ± 8.67	-
62	R1-2B/J804 B	WU35	81.44 ± 1.05	+++
63	R1-2B/M201		76.88 ± 3.36	++
64	R1-2B/N201		27.25 ± 7.71	-
65	R1-2B/N202		9.31 ± 4.43	-
66	R1-2B/N204		25.43 ± 4.35	-
67	R1-2B/N205	WU32	80.71 ± 1.39	++
68	R1-2B/N707	WU33	84.35 ± 0.80	+++

No	Sample code	WU code	% Inhibition at 0.5 mg/ml	Antimalarial activity
69	R1-2B/O202		43.92 ± 0.10	-
70	R1-3/B410	WU34	84.68 ± 1.95	+++
71	R1-3/B412 A		24.97 ± 2.09	-
72	R1-3/D102		5.02 ± 6.97	-
73	R1-3/H103		48.15 ± 0.40	-
74	R1-3/H401		75.50 ± 1.55	++
75	R1-3/H401 B		74.31 ± 5.14	++
76	R1-3/H404		29.19 ± 1.88	-
77	R1-3/K306		76.29 ± 3.30	++
78	R1-5		32.23 ± 7.85	-
79	R1-37		47.16 ± 4.35	-
80	R1-A106		31.84 ± 2.10	-
81	R1-E3		39.50 ± 3.39	-
82	R2-M301		5.75 ± 7.59	-
83	R2-N501		75.76 ± 1.52	++
84	R3-L301		14.13 ± 6.47	-
85	R3-N303		14.49 ± 5.19	-
86	R3-N304		71.92 ± 1.85	++
87	R3-N401		9.41 ± 1.64	-
88	R3-S503		1.25 ± 4.29	-
89	R4-D401		65.24 ± 7.70	+
90	R5-D204		48.15 ± 9.69	-
91	R5-L305		66.08 ± 2.73	+

No	Sample code	WU code	% Inhibition at 0.5 mg/ml	Antimalarial activity
92	R5-M301		14.17 ± 6.50	-
93	R6-J501		26.12 ± 2.52	-
94	R7-K303		57.73 ± 0.45	+
95	R7-M101 A		13.72 ± 1.45	-
96	R7-M301 B		20.75 ± 3.95	-
97	R7-N305 A		2.22 ± 1.78	-
98	R7-N306		7.36 ± 2.08	-
99	R8-J107		0.67 ± 4.85	-
100	R8-K310 B		2.47 ± 5.51	-
101	R8-K405		0.32 ± 2.32	-
102	R8-K405 B		10.89 ± 8.88	-
103	R8-N301		31.26 ± 0.55	-
104	R8-S107		36.20 ± 8.69	-
105	Rh1-5-9		3.50 ± 1.86	-
106	Rh1-5-10	WU14	21.49 ± 6.36	-
107	Rh1-5-11		20.08 ± 6.28	-
108	Rh1-5-13		25.86 ± 5.50	-
109	Rh1-5-14	WU17	38.58 ± 2.95	-
110	Rh1-5-22	WU21	12.24 ± 2.30	-
111	Rh1-6-2		7.81 ± 2.12	-
112	S3-31		18.34 ± 1.09	-
113	S6-11		33.08 ± 1.01	-
114	S6-14		32.68 ± 4.66	-

No	Sample code	WU code	% Inhibition at 0.5 mg/ml	Antimalarial activity
115	S6-28		20.14 ± 3.60	-
116	S6-31		53.87 ± 6.25	+
117	S13-1		2.98 ± 13.69	-
118	S13-2		23.85 ± 6.73	-
119	S32-63		40.29 ± 2.88	-
120	S32-76		49.12 ± 4.78	-
121	S32-77		38.32 ± 0.94	-
122	S32-79		57.54 ± 4.80	+
123	S33-1		1.09 ± 1.54	-
124	S54-15		42.60 ± 11.36	-
125	S54-18		9.85 ± 5.66	-
126	S1-SC1	WU20	57.95 ± 2.86	+
127	S1-SC3	WU22	69.50 ± 1.71	++
128	S1-SC13	WU23	59.17 ± 1.71	+
129	S2-SC1		14.13 ± 2.02	-
130	S2-SC2	WU28	38.15 ± 5.91	-
131	S2-SC4		31.59 ± 4.12	-
132	S2-SC6		17.80 ± 4.86	-
133	S2-SC7		18.17 ± 2.16	-
134	S2-SC9		9.38 ± 4.84	-
135	S2-SC10	WU18	65.35 ± 3.74	+
136	S2-SC12		2.51 ± 4.04	-
137	S2-SC13		27.34 ± 0.43	-

No	Sample code	WU code	% Inhibition at 0.5 mg/ml	Antimalarial activity
138	S2-SC14		0.14 ± 2.39	-
139	S2-SC16	WU13	53.33 ± 6.19	+
140	S2-SC19	WU05	91.38 ± 0.29	+++
141	S2-SC24		65.02 ± 2.10	+
142	S2-SC30		78.81 ± 2.77	++
143	S2-SC32	WU27	19.70 ± 5.01	-
144	S2-SC33		15.51 ± 5.53	-
145	S2-SC38	WU11	80.53 ± 2.41	+++
146	S2-SC40		79.25 ± 2.07	++
147	S4-SC1		0.79 ± 2.18	-
148	S4-SC5		39.40 ± 3.91	-
149	S4-SC9		56.31 ± 4.33	+
150	S4-SC11	WU10	84.96 ± 3.18	+++
151	S5-SC2	WU04	81.28 ± 1.51	+++
152	S5-SC5	WU06	88.31 ± 0.77	+++
153	S5-SC6	WU07	88.26 ± 6.04	+++
154	S5-SC11		8.01 ± 4.96	-
155	S5-SC13		66.56 ± 3.07	+
156	S5-SC14	WU09	100.98 ± 2.35	+++
157	S6-SC2	WU12	83.42 ± 2.50	+++
158	S6-SC3		31.02 ± 2.88	-
159	S6-SC6		19.80 ± 7.24	-
160	S7-SC6		53.47 ± 3.27	+

No	Sample code	WU code	% Inhibition at 0.5 mg/ml	Antimalarial activity
161	S7-SC8		38.73 $\pm$ 6.82	-
162	S7-SC9	WU08	87.70 $\pm$ 4.70	+++
163	S7-SC10		25.15 $\pm$ 3.82	-
164	S7-SC18		79.65 $\pm$ 4.30	++

Antimalarial activity, +++ ; +++; +;+++; active , ++; active, +; inactive?

Table S2 A list of annotated compounds obtained from MS positive and negative mode analysis in clusters using GNPS

**Hits**	**Exact mass**	**Formula**	**[M+H]⁺; *m/z***	**[M-H]⁻; *m/z***	**Shared peaks**	**Strains**	**Rt (min)**	**Bioactivities**	**Reference**
Chymostatin B (**1**)	593.296	$C_{30}H_{39}N_7O_6$	$[M+H]^+$; 594.302		15	S7-SC9	7.645	Enzyme inhibitors	(1)
Geldanamycin (**2**)	560.273	$C_{29}H_{40}N_2O_9$	$[M+Na]^+$; 583.263	$[M-H]^-$; 559.260	17	S2-SC19, 1-3	10.095	Antimalarial activity (20 nM); anti-Hsp90[a,b]	(2, 3)
Dehydroxynocard-amine (**3**)	584.715	$C_{27}H_{48}N_6O_8$	$[M+H]^+$; 585.361	$[M-H]^-$; 583.340	25	S2-SC19, 1-3, S4-SC11	5.997	Antimalarial activity (4.5 µM)	(4, 3)
Elaiophylin (**4**)	1025.270	$C_{54}H_{88}O_{18}$	$[M+ Na]^+$; 1047.580	$[M-H]^-$; 1023.580	22	S2-SC19, 1-3	12.196	Antimalarial activity (0.78 µM)	(5, 3)
Ferrioxamine B (**5**)	613.260	$C_{25}H_{48}N_6O_{12}Fe$	$[M+H]^+$; 614.270		19	S4-SC11	4.694	Siderophore transporter	(6)
Desferrioxamine D2 (**6**)	586.333	$C_{26}H_{46}N_6O_9$	$[M+Na]^+$; 609.322	$[M-H]^-$; 585.319	25	S2-SC19, 1-3	5.923	Siderophore transporter	(6)

**Hits**	**Exact mass**	**Formula**	**[M+H]$^+$; *m/z***	**[M-H]$^-$; *m/z***	**Shared peaks**	**Strains**	**Rt (min)**	**Bioactivities**	**Reference**
Ikarugamycin epoxide (**7**)	494.632	$C_{29}H_{38}N_2O_5$	[M+H]$^+$; 495.285		19	S5-SC2	10.987	Antibacterial and anti-tumor activities	(7)
Kanchanamycin C (**8**)	1053.630	$C_{54}H_{91}N_3O_{17}$	[M+H]$^+$; 1054.640	[M-H]$^-$; 1052.620	39	S2-SC19, 1-3	8.656	Antibacterial activity	(8)
Glochidone (**9**)	422.697	$C_{30}H_{46}O$	[M+H]$^+$; 423.362		15	S4-SC11	9.078	Antibacterial activity	(9)
Bisucaberin (**10**)	400.476	$C_{18}H_{32}N_4O_6$	[M+H]$^+$; 401.239		13	S2-SC19, 1-3, S4-SC11	5.624	Anticancer activity	(10, 11)
Desferrioxamine G (**11**)	618.359	$C_{27}H_{50}N_6O_{10}$	[M+H]$^+$; 619.366		15	S2-SC19, 1-3	4.986	Siderophore transporter	(6)
Deferrioxamine E (**12**)	600.348	$C_{27}H_{48}N_6O_9$	[M+Na]$^+$; 623.337	[M-H]$^-$; 599.334	26	S2-SC19, 1-3, S4-SC11	6.150	Siderophore transporter	(6)
Desferrioxamine (**13**)	560.684	$C_{25}H_{48}N_6O_8$	[M+H]$^+$; 561.361		14	S4-SC11	5.231	Antimlarial activity (6 μM); anti-HIV activity	(12, 13, 14)

**Hits**	**Exact mass**	**Formula**	**[M+H]⁺; *m/z***	**[M-H]⁻; *m/z***	**Shared peaks**	**Strains**	**Rt (min)**	**Bioactivities**	**Reference**
Desferrioxamine H (**14**)	575.317	$C_{26}H_{46}N_6O_9$	$[M+Na]^+$; 576.324		18	S4-SC11	6.056	Siderophore transporter	(6)
Ferrioxamine E (**15**)	653.535	$C_{27}H_{45}FeN_6O_9$	$[M+H]^+$; 654.268	$[M-H]^-$; 635.310	7	S2-SC19, 1-3		Siderophore transporter	(6)
Coproporphyrin III (**16**)	654.720	$C_{36}H_{38}N_4O_8$		$[M-H]^-$; 653.255	26	S4-SC11		Antifungal activity	(15)
Futalosine (**17**)	414.374	$C_{19}H_{18}N_4O_7$		$[M-H]^-$; 413.105	8	S2-SC19, 1-3, S4-SC11, S5-SC2		Antibacterial activity	(16)

a: antimalarial activity; b: anti-HIV activity

Table S3 ^{1}H NMR spectra of isolated compound and compound **4** (500 MHz, MeOD)

**Our NMR experiment**				**NMR experiment of Cao *et al*. (17)**					
**^{1}H NMR (ppm)**	**Hz**	**Number of protons**	**Proton assignments**	**^{1}H NMR (ppm)**	**Hz**	**Number of H**	**H assignments**	**^{13}C NMR (ppm)**	**Position**
6.95 (t)	J=15	1H	H-3	6.93 (dd)	J = 15.2, 11.2	1H	H-3	170.4	C-1
6.19 (dd)	J=15, J=10	1H	H-4	6.17 (dd)	J = 15.0, 11.2	1H	H-4	146.9	C-3
5.76 (d)	J=15	1H	H-2	5.74 (d)	J = 15.4	1H	H-2	146.1	C-5
5.67 (m)		1H	H-5	5.67 (dd)	J = 15.1, 9.9	1H	H-5	132.7	C-4
5.06 (m)		2H	H-7, H-22	5.04 (m)		2H	H-7, H-22	122.6	C-2
4.03 (d)	J=10	1H	H-13	4.02 (d)	J = 9.7	1H	H-13	100.9	C-11
3.94 (m)		4H	H-24, H-26, H-9, H-15	3.94 (m)		2H	H-24, H26	94.8	C-22
				3.9 (m)		2H	H-9, H-15	78.2	C-7
3.54 (d)	J=10	1H	H-25	3.53 (m)		1H	H-25	72.4	C-25
2.60 (m)		1H	H-26	2.58 (m)		1H	H-26	71.8	C-9

**Our NMR experiment**				**NMR experiment of Cao *et al*. (17)**					
**^{1}H NMR (ppm)**	**Hz**	**Number of protons**	**Proton assignments**	**^{1}H NMR (ppm)**	**Hz**	**Number of H**	**H assignments**	**^{13}C NMR (ppm)**	**Position**
2.36 (dd)	J=5, J=10	1H	H-12	2.34 (dd)	J = 12.1, 4.5	1H	H-12	70.9	C-13
1.94 (m)		3H	H-2, H23	1.95 (m)		1H	H-8	68.2	C-15
				1.94 (dd)	J = 12.3, 3.9	1H	H-23	68.1	C-26
1.72 (m)		1H	H-10	1.72 (m)		1H	H-10	67	C-24
1.64 (m)		2H	H-20, H23	1.66 (m)		1H	H-20	49.8	C-14
				1.62 (dd)	J = 12.6, 4.7	1H	H-23	44	C-10
1.47 (m)		2H	H-20	1.47 (m)		1H	H-20	42.7	C-6
1.21 (d)	J=5	3H	H-17	1.2 (d)	J = 6.6	3H	H-17	38.9	C-12
1.17 (m)		1H	H-16	1.16 (m)		1H	H-16	37.8	C-8
1.14 (d)	J=10	3H	H-16	1.13 (d)	J = 6.0	3H	H-16	33.7	C-23
1.12 (m)		1H	H-12	1.12 (m)		1H	H-12		
1.06 (d)	J=5	3H	H-17	1.05 (d)	J = 6.6	3H	H-17		
0.99 (d)	J=10	3H	H-19	0.97 (d)	J = 7.1	3H	H-19		

Our NMR experiment				NMR experiment of Cao *et al*. (17)					
**^{1}H NMR (ppm)**	**Hz**	**Number of protons**	**Proton assignments**	**^{1}H NMR (ppm)**	**Hz**	**Number of H**	**H assignments**	**^{13}C NMR (ppm)**	**Position**
0.89 (m)		6H	H-21, H-18	0.87 (t)	J = 6.2	6H	H-21, H-18		

Table S4 Whole genome sequence summary of the strain S2-SC19

**Parameters**	**Amount**
Depth of coverage	71 ×
Total reads	3.349 M
Genome size (bp)	10,585,008
% G+C	71.44
Number of contigs	237
N50	88,717
Completeness	99.47%
Contamination	1.84%
Genome-based taxonomy	*Streptomyces asiaticus*
Gene	8,869
CDS	8,775
rRNA genes	6
tRNA genes	87
tmRNA genes	1
Antimicrobial resistance genes	Not found
2nd metabolite biosynthesis genes	Yes
Single-copy gene clusters	3,676
Total variant	146,769

Table S5 Putative biosynthetic gene clusters of the actinobacteria strain S2-SC19 based on genome analysis by antiSMASH

**BGC**	**Type**	**Putative metabolite**
1.1	PKS-like	rustmicin
1.2	terpene	2-methylisoborneol
1.3	PKS type I	geldanamycin
2.1	butyrolactone	oryzanaphthopyran A, B, and C/ oryzanthrone A and B/ chlororyzanthrone A and B
2.2	hserlactone	heronamide A, B, C, D, E, and F
3.1	NRPS	
4.1	hydrogen-cyanide	aborycin
6.1	PKS type I	neomediomycin B
8.1	NI-siderophore	peucechelin
8.2	butyrolactone	cyphomycin
9.1	terpene	brasilicardin A
9.2	NRPS	cyclofaulknamycin
10.1	NRPS-like	echoside A, B, C, D, and E
10.2	betalactone	Sch-47554/Sch-47555
11.1	PKS type I	desulfoclethramycin/clethramycin
14.1	terpene	pristinol
15.1	lanthipeptide class II	reveromycin A
17.1	PKS type I	salinomycin
20.1	NRPS	ochronotic pigment
21.1	NI-siderophore	legonoxamine A and B/desferrioxamine B
22.1	terpene	TVA-YJ-2
25.1	indole	5-isoprenylindole-3-carboxylate ß-D-glycosyl ester
27.1	PKS type I	nigericin

BGC	Type	Putative metabolite
27.2	PKS type I, NRPS, betalactone	elaiophylin
30.1	terpene	hopene
32.1	PKS type I	cyphomycin
34.1	NRPS	corbomycin
35.1	arylpolyene	kitacinnamycin A, B, C, D, E, and F
36.1	RiPP-like	
36.2	PKS type I	s56-p1
39.1	NRPS-like	echosides
41.1	NRPS-like, NRPS	glycinocin A
43.1	NI-siderophore	peucechelin
47.1	NAPAA	ε-Poly-L-lysine
50.1	redox-cofactor	lankacidin C
50.2	PKS type I	efomycin K and L
56.1	ectoine	ectoine
58.1	RiPP-like, PKS type I, hglE-KS	hexacosalactone A
61.1	PKS type II	WS79089A/hexaricin B and C
63.1	PKS type I, NRPS-like	streptolydigin
64.1	arylpolyene	cinnapeptin
65.1	NRPS-like	hygrocin A and B
65.2	PKS type I	meridamycin
66.1	PKS type I	efomycin K and L
67.1	NI-siderophore	kinamycin
68.1	NRPS	

BGC	Type	Putative metabolite
69.1	PKS type I	hexacosalactone A
73.1	terpene	
75.1	NRPS	glycinocin A
77.1	PKS type I	notonesomycin A
79.1	terpene	aurachin C, D and SS
85.1	RiPP-like	granaticin
88.1	NRPS, PKS type I	meridamycin
95.1	NRPS	skyllamycin D and E
107.1	PKS type III, NRPS, other	feglymycin
108.1	PKS type II	spore pigment
110.1	PKS type I	tautomycin
113.1	PKS type I	maklamicin
116.1	PKS type III, NRPS-like, terpene	totopotensamide A and B
125.1	PKS type I	ebelactone
126.1	PKS type I	lobophorin A
127.1	PKS type I	olimycin A and B
131.1	PKS type I	nigericin
133.1	PKS type I	azalomycin F3a
135.1	PKS type I	niphimycin C-E
138.1	PKS type I	ECO-0501
142.1	PKS type I	
145.1	PKS type I	
147.1	PKS type I	mediomycin A
148.1	PKS type I	candicidin

BGC	Type	Putative metabolite
149.1	PKS type I	neomediomycin B
150.1	PKS type I	
152.1	PKS type I	desulfoclethramycin/clethramycin
156.1	PKS type I	linearmycin A, B, and C
162.1	PKS type I	FD-891
166.1	NRPS	actinomycin D
168.1	PKS type I	aculeximycin
170.1	NRPS	
171.1	PKS type I	tetrafibricin
173.1	PKS type I	
176.1	PKS type I	nocamycin V
177.1	PKS type I	thaxteramide C
178.1	PKS type I	
179.1	PKS type I	
180.1	PKS type I	ECO-02301
183.1	PKS type I	
187.1	NRPS	
188.1	PKS type I	
192.1	NRPS	
193.1	NRPS	marformycin A, B, C, D, E, and F
196.1	PKS type I	neomediomycin B
197.1	PKS type I	
204.1	PKS type I	
205.1	PKS type I	quinolidomicin A
207.1	NRPS	

BGC	Type	Putative metabolite
208.1	PKS type I	
213.1	PKS type I	
216.1	NRPS	rhizomide A, B, and C
217.1	PKS type I	
218.1	PKS type I	
219.1	NRPS	
221.1	PKS type I	
222.1	PKS type I	

Table S6 Putative biosynthetic gene clusters coding for elaiophylin-like compounds in actinobacteria strain S2-SC19 based on genome analysis by antiSMASH

**Contig**	**Region**	**Type**	**From**	**To**	**Most similar known BGC**	**Type**	**Similarity**
Contig 27	Region 2	PKS type I, NRPS, betalactone	29,886	100,986	elaiophylin	polyketide	45%
Contig 50	Region 2	PKS type I	56,057	78,648	efomycin K, efomycin L	polyketide	37%
Contig 66	Region 1	PKS type I	27,127	61,591	efomycin K, efomycin L	polyketide	66%
Contig 133	Region 1	PKS type I	1	20,369	azalomycin F3a	polyketide	39%

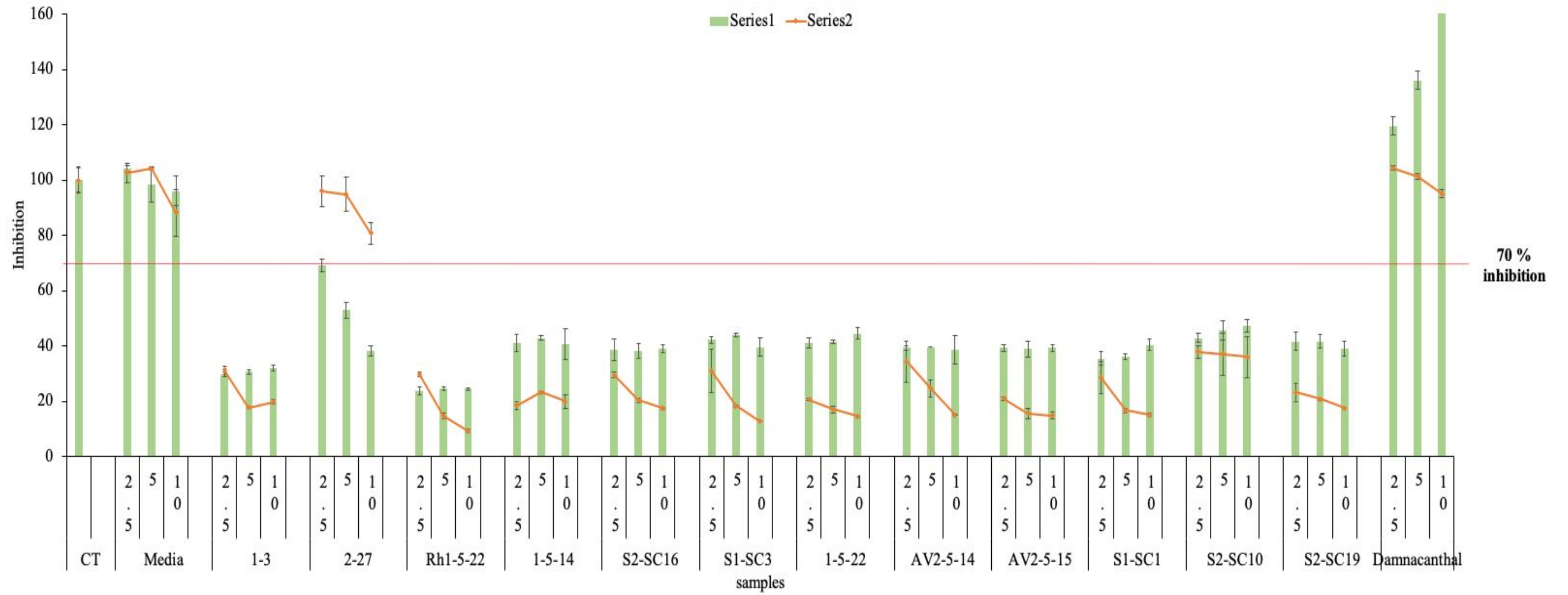

Figure S1 A bar chart showing the antiviral activity (green) and the cytotoxicity (orange) of Thai actinobacterial isolates (final concentrations ranged from 2.5 to 10 μg/mL)

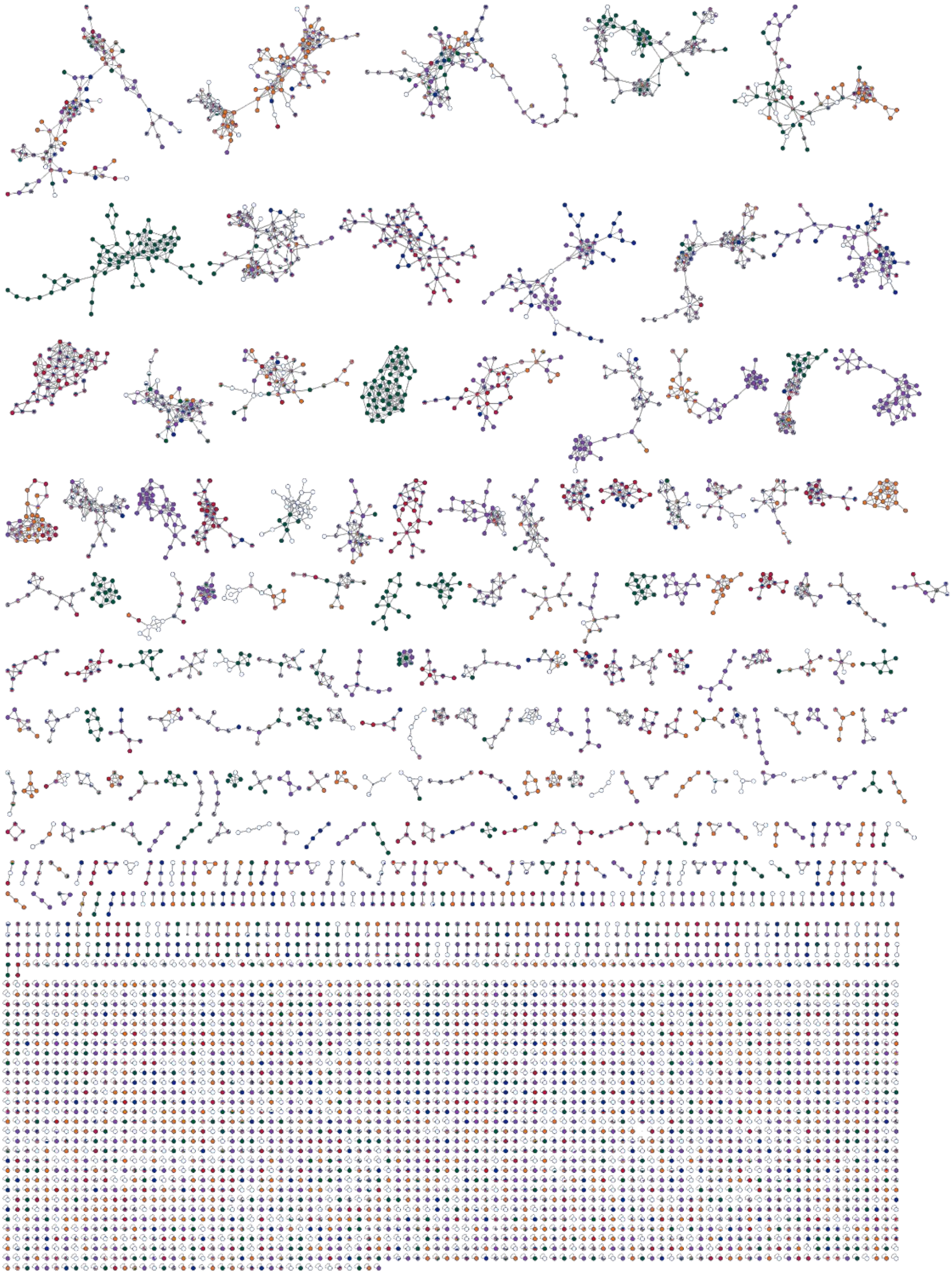

Figure S2 Bioactive molecular network in positive mode of selected Thai actinobacterial crude extracts. Node colors: red = S2-SC19; blue = 1-3; Violet = S4-SC11; orange = S5-SC2; green = S7-SC9.

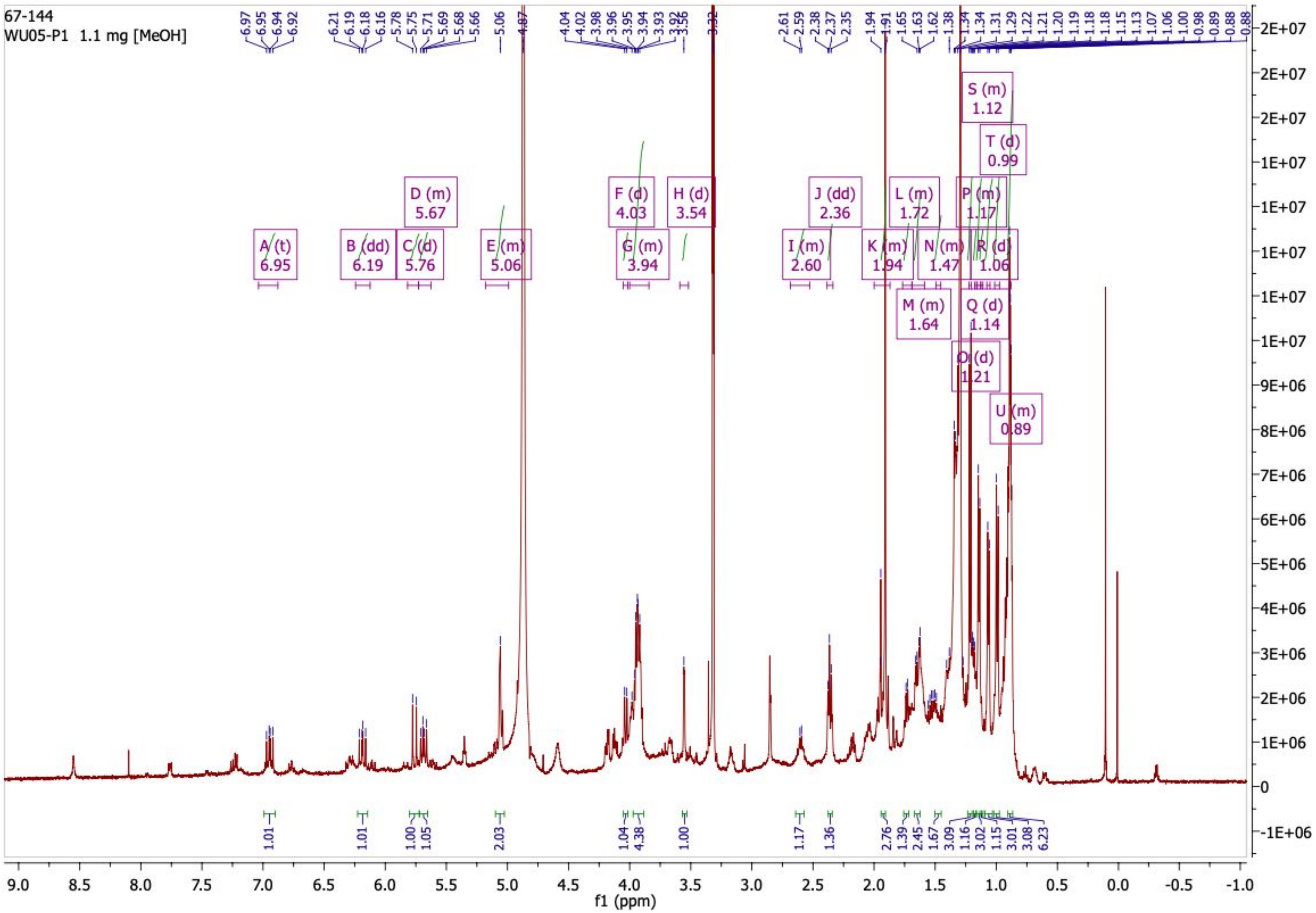

Figure S3 ^{1}H NMR spectrum of the isolated compound (MeOD, 500 MHz)

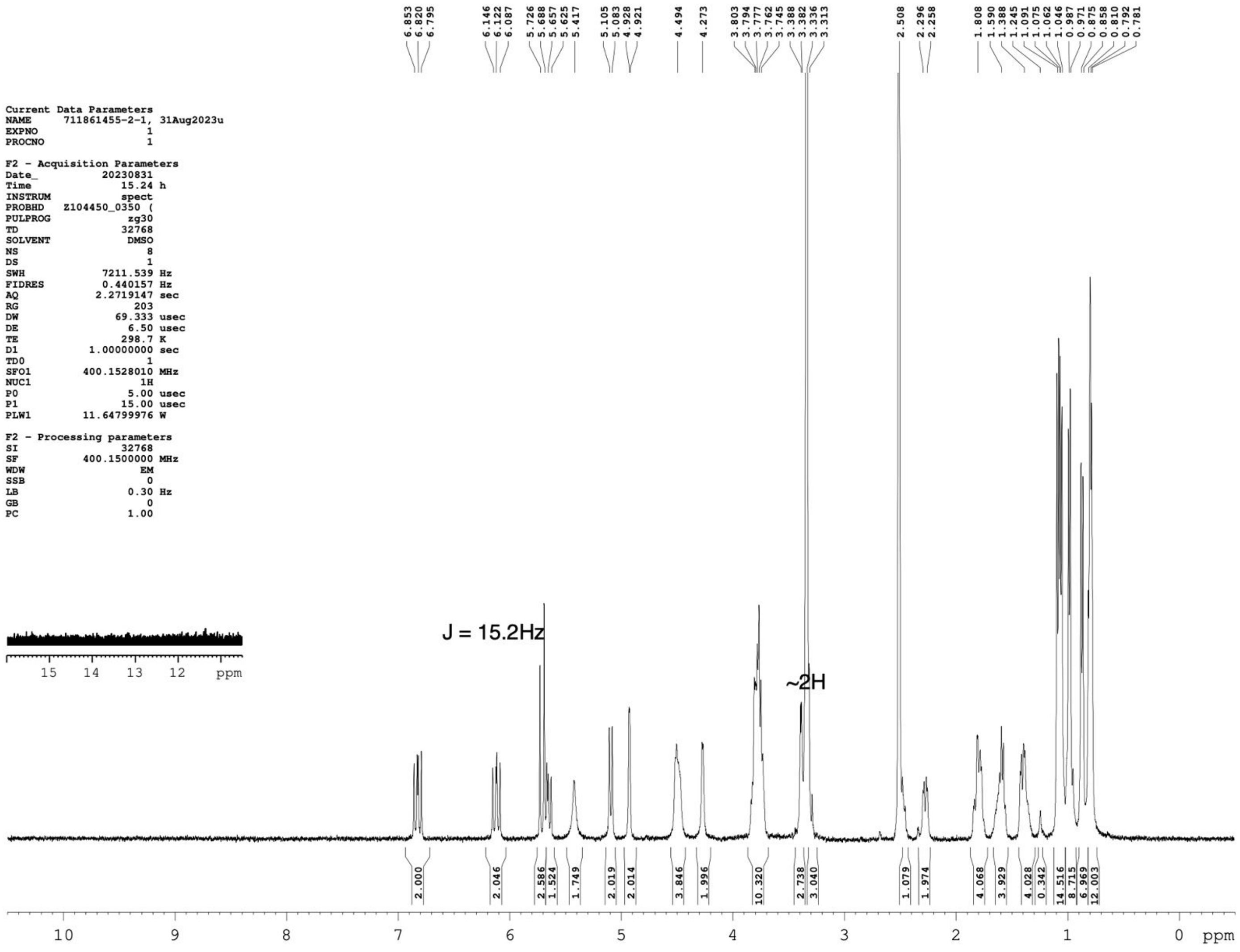

Figure S4 ^{1}H NMR spectrum of the purchased 85% purity elaiophylin (DMSO-d6, 400 MHz) (Toronto Research Chemicals, Canada)

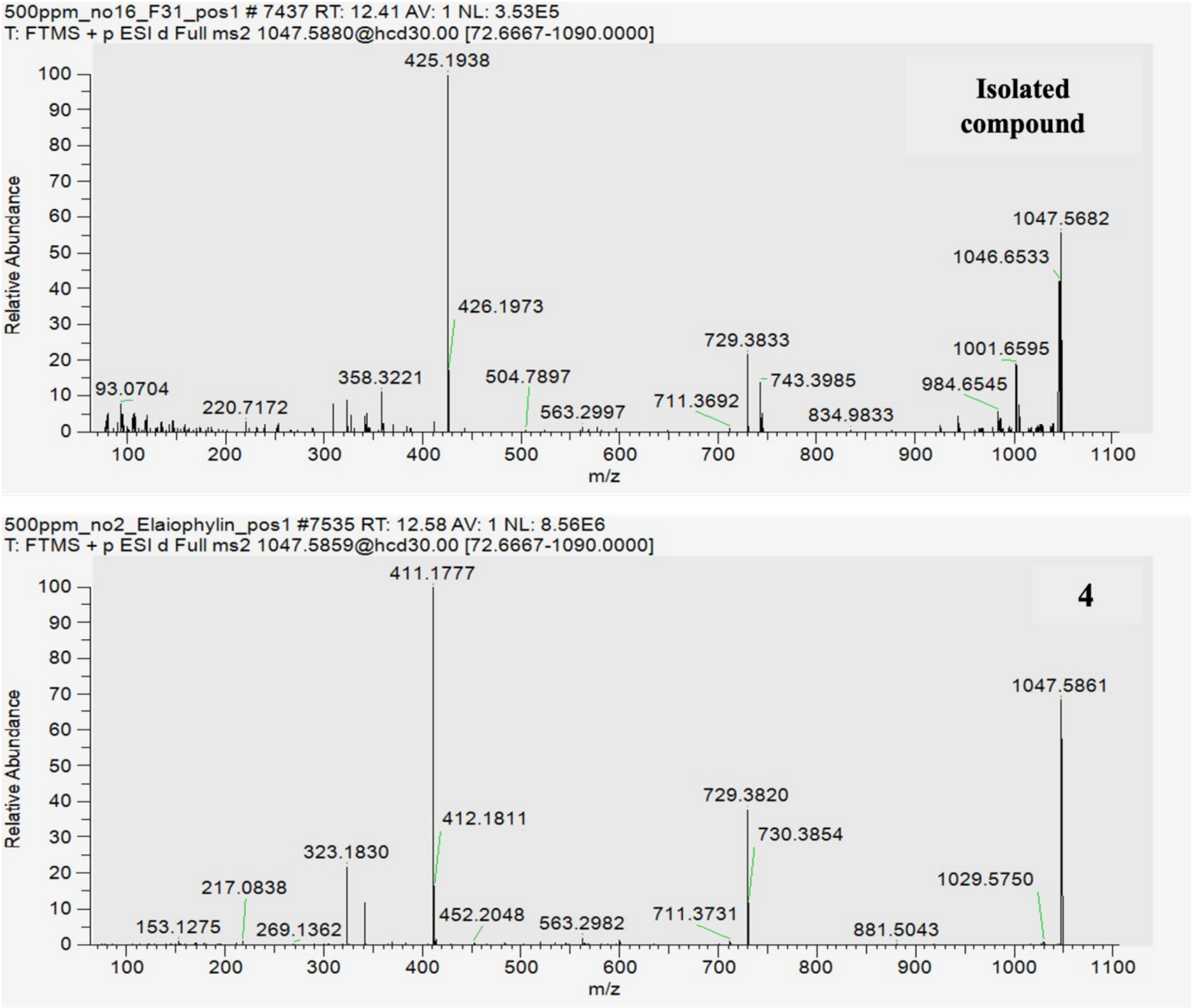

Figure S5 LCMS fragment picture of the isolated compound and **4**

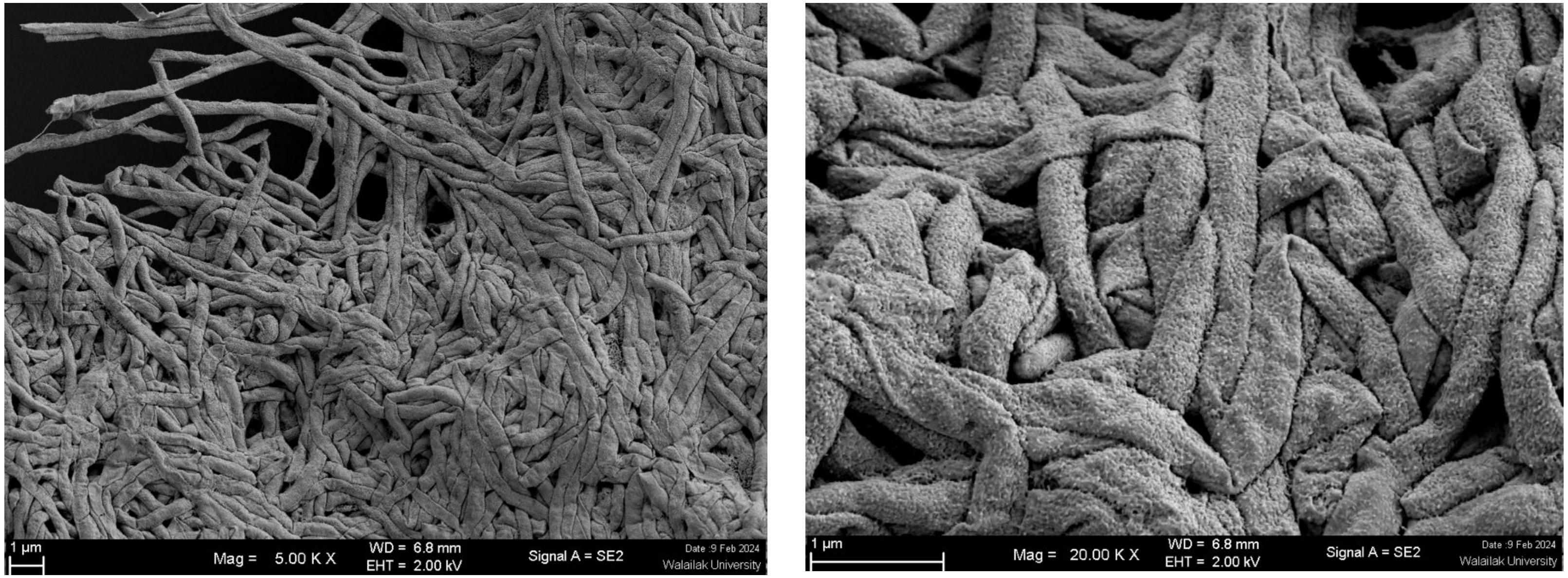

Figure S6 Scanning electron micrographs of cells of strain S2-SC19 after growth in ISP 2 broth for 3 days at 28 °C. Bars, 1 μm

Reference

(1) Tatsuta, K.; Mikami, N.; Fujimoto, K., Umezawa; S.; Umezawa, H. The structure of chymostatin, a chymotrypsin inhibitor. *J. Antibiot*. **1973**, *26*(11), 625-46. https://doi.org/10.7164/antibiotics.26.625.

(2) Kitson, R.R.A.; Kitsonová, D.; Siegel, D.; Ross, D.; Moody, C.J. Geldanamycin, a naturally occurring inhibitor of Hsp90 and a lead compound for medicinal chemistry. *J. Med. Chem.* **2024**, *67*, 17946–63. https://doi.org/10.1021/acs.jmedchem.4c01048.

(3) Happi, G.M.; Ntabo, V.K.; Soh, D.; Wansi, J.D. Potential of *Streptomyces* in producing antiplasmodial lead compounds. *Natural Resources for Human Health*. **2023**, *3*(1), 7-20. https://doi.org/10.53365/nrfhh/150397.

(4) Shaaban, K.; Singh, S.; Elshahawi, S.; Wang, X.; Ponomareva, L.V.; Sunkara, M.; Copley, G.C.; Hower, J.C.; Morris, A.J.; Kharel, M.K.; Thorson, J.S. Venturicidin C, a new 20-membered macrolide produced by *Streptomyces* sp. TS-2-2. *J. Antibiot.* **2014**, *67*, 223–230. https://doi.org/10.1038/ja.2013.113.

(5) Sheng, Y.; Lam, P.W.; Shahab, S.; Santosa, D.A.; Proteau, P.J.; Zabriskie, T.M.; Mahmud, T. Identification of elaiophylin skeletal variants from the Indonesian *Streptomyces* sp. ICBB 9297. *J. Nat. Prod.* **2015**, *78*(11), 2768–75. https://doi.org/10.1021/acs.jnatprod.5b00752.

(6) Müller, G.; Raymond, K.N.; Specificity and mechanism of ferrioxamine-mediated iron transport in *Streptomyces pilosus*. *J. Bacteriol.* **1984**, *160*(1), 304-12. https://doi.org/10.1128/jb.160.1.304-312.1984. PMID: 6480557; PMCID: PMC214717.

(7) Bertasso, M.; Holzenkaempfer, M.; Zeeck, A.; Stackebrandt, E.; Beil, W.; Fiedler, H.P. Ripromycin and other polycyclic macrolactams from *Streptomyces* sp. Tü 6239 taxonomy,

fermentation, isolation and biological properties. *J. Antibiot.* **2003**, *56*(4), 364-371. https://doi.org/10.7164/antibiotics.56.364.

(8) Ubukata, M.; Morita; T.; Osada, H. RS-22A, B and C: new macrolide antibiotics from *Streptomyces violaceusniger*. II. Physico-chemical properties and structure elucidation. *J. Antibiot*. **1995**, *48*(4):293-9. https://doi.org/10.7164/antibiotics.48.293.

(9) Shaikh, A.; Nothias, L.-F.; Srivastava, S.; Dorrestein, P.; Tahlan, K. Specialized metabolites from ribosome engineered strains of *Streptomyces clavuligerus*. *Metabolites*. **2021**, *11*,239. https://doi.org/10.3390/metabo11040239.

(10) Liu, J.; Nothias, L.F.; Dorrestein, P.C.; Tahlan, K., Bignell, D.R.D. Genomic and metabolomic analysis of the potato common scab pathogen *Streptomyces scabiei*. *ACS Omega.* **2021**, *6*(17), 11474-11487. https://doi.org/10.1021/acsomega.1c00526.

(11) Miller, S.J.; Morgan, B.W. *Deferoxamine*. *In Encyclopedia of Toxicology (Third Edition)*; Wexler, P., Ed.; Oxford: Academic Press, **2014**, 1154–56. https://doi.org/10.1016/B978-0-12-386454-3.00721-1.

(12) Kameyama, T.; Takahashi, A.; Kurasawa, S.; Ishizuka, M.; Okami, Y.; Takeuchi, T.; Umezawa, H. (1987). Bisucaberin, a new siderophore, sensitizing tumor cells to macrophage-mediated cytolysis. I. Taxonomy of the producing organism, isolation and biological properties. *J. Antibiot.* **1987**, *40*(12), 1664–1670. https://doi.org/10.7164/antibiotics.40.1664

(13) Scott, M.D.; Ranz, A.; Kuypers, F.A.; Lubin, B.H.; Meshnick, S.R. Parasite uptake of desferroxamine: a prerequisite for antimalarial activity. *Br. J. Haematol.* **1990**, *75*(4), 598–602. https://doi.org/10.1111/j.1365-2141.1990.tb07805.x.

(14) Debebe, Z.; Ammosova, T.; Jerebtsova, M.; Kurantsin-Mills, J.; Niu, X.; Charles, S.; Richardson, D.R.; Ray, P.E.; Gordeuk, V.R.; Nekhai, S. Iron chelators ICL670 and 311 inhibit HIV-1 transcription. *Virol.* **2007**, *367*(2), 324–333. https://doi.org/10.1016/j.virol.2007.06.011.

(15) Perez-Ortiz, G.; Sidda, J.D.; Peate, J.; Ciccarelli, D.; Ding, Y.; Barry, S.M. Production of copropophyrin III, biliverdin and bilirubin by the rufomycin producer, *Streptomyces atratus*. *Front. Microbiol.* **2023**, *14*,1092166. https://doi.org/10.3389/fmicb.2023.1092166.

(16) Ogasawara, Y.; Kondo, K.; Ikeda, A.; Harada, R.; Dairi, T. Identification of tirandamycins as specific inhibitors of the futalosine pathway. *J. Antibiot.* **2017**, *70*, 798–800. https://doi.org/10.1038/ja.2017.22.

(17) Cao, P.; Li, C.; Wang, H.; Yu, Z.; Xu, X.; Wang, X.; Zhao, J.; Xiang, W. Community structures and antifungal activity of root-associated endophytic Actinobacteria in healthy and diseased cucumber plants and *Streptomyces* sp. HAAG3-15 as a promising biocontrol agent. *Microorganisms*. **2020**, *8*(2), 236. https://doi.org/10.3390/microorganisms8020236
